# Comparative metabolic profiling of the lipid-producing green microalga *Chlorella* reveals that nitrogen and carbon metabolic pathways contribute to lipid metabolism

**DOI:** 10.1186/s13068-017-0839-4

**Published:** 2017-06-15

**Authors:** Hui Chen, Yanli Zheng, Jiao Zhan, Chenliu He, Qiang Wang

**Affiliations:** 1 Key Laboratory of Algal Biology, Institute of Hydrobiology, The Chinese Academy of Sciences, Wuhan, 430072 Hubei China; 2 University of the Chinese Academy of Sciences, Beijing, 100039 China; 3 State Key Laboratory of Freshwater Ecology and Biotechnology, Institute of Hydrobiology, The Chinese Academy of Sciences, Wuhan, 430072 Hubei China

**Keywords:** Carbon metabolism, Lipid metabolism, Metabolome, Microalgae, Nitrogen assimilation, Nitrogen salvage

## Abstract

Microalgae are a promising feedstock for biofuel production. Microalgal metabolic pathways are heavily influenced by environmental factors. For instance, lipid metabolism can be induced by nitrogen-limiting conditions. However, the underlying mechanisms of lipid biosynthesis are unclear. In this study, we analyzed the global metabolic profiles of three genetically closely related *Chlorella* strains (C1, C2, and C3) with significant differences in lipid productivity to identify the contributions of key metabolic pathways to lipid metabolism. We found that nitrogen obtained from amino acid catabolism was assimilated via the glutamate–glutamine pathway and then stored as amino acids and intermediate molecules (particularly proline, alanine, arginine, succinate, and gamma-aminobutyrate) via the corresponding metabolic pathways, which led to carbon–nitrogen disequilibrium. Excess carbon obtained from photosynthesis or glycolysis was re-distributed into carbon-containing compounds, such as glucose-6-phosphate, fructose-6-phosphate, phosphoenolpyruvate, lactate, citrate, 3-hydroxybutyrate, and leucine, and then diverted into lipid metabolism for the production of storage lipids via the gamma-aminobutyrate pathway, glycolysis, and the tricarboxylic acid cycle. These results were substantiated in the model green alga *Chlamydomonas reinhardtii* by analyzing various mutants deficient in glutamate synthase/NADH-dependent, glutamate synthase/Fd-dependent, glutamine synthetase, aspartate aminotransferase, alanine aminotransferase, pyruvate kinase, and citrate synthase. Our study suggests that not only carbon but also nitrogen assimilation and distribution pathways contribute to lipid biosynthesis. Furthermore, these findings may facilitate genetic engineering efforts to enhance microalgal biofuel production.

**Electronic supplementary material:**

The online version of this article (doi:10.1186/s13068-017-0839-4) contains supplementary material, which is available to authorized users.

## Background

Biodiesel, one of the most commonly used biofuels, has attracted much attention as an ideal renewable energy carrier, and thus also as a possible primary energy source [1]. Recently, there has been renewed interest in producing biodiesel from microalgae, as microalgae can grow rapidly and convert solar energy into chemical energy via CO_2_ fixation. Microalgae are thus now considered to be one of the most promising sources of oil for biodiesel production [2, 3].

Using microalgae for environmental purposes (CO_2_ fixation, NOx, and wastewater treatment) has also attracted much attention recently, and some environmental factors could induce lipid accumulation [4–6]. Microalgal metabolic pathways are heavily influenced by the environment [7]. Nitrogen (N) limitation and N starvation are primary factors that influence cell growth and metabolism, and these conditions may be used to enhance lipid biosynthesis in microalgae. When N is insufficient to support protein synthesis, excess carbon (C) from photosynthesis is diverted into storage molecules such as triglycerides and starch [8].

The underlying mechanisms of lipid biosynthesis in microalgae are unknown and many questions remain about the basic biology of microalgae, particularly the metabolic changes induced by environmental stress. Targeted proteomics, metabolomics, and metabolic flux analyses have been performed on *Chlamydomonas reinhardtii* grown under mixotrophic and autotrophic conditions [9]. The growth and development of plants and algae depends on N supply and assimilation. Algae have evolved a variety of N assimilation pathways to make use of the variety of N forms (N_2_, NH_4_^+^, NO_3_^−^, NO_2_^−^, and dissolved organic N) present in the environment [10]. Assimilation of these various forms of N requires a number of complex enzymes [11]. Abiotic stress often causes amino acids, which serve as potential stress mitigators, to accumulate [12]. In addition to being the building blocks of proteins, amino acids serve as the precursors of N-containing molecules such as nucleic acids, polyamines, quaternary ammonium compounds, and some hormones. Under environmental stress, de novo protein synthesis is generally inhibited and protein turnover and proteolytic activity are increased, resulting in an increase of total free amino acids [13, 14]. N and C metabolisms are closely connected; N assimilation and amino acid biosynthesis require reducing equivalents from photosynthesis and C skeletons from the tricarboxylic acid (TCA) cycle [15, 16].

The green microalga *Chlorella* (Chlorophyta), which includes about 10 species, can grow photoautotrophically, mixotrophically, and heterotrophically with high biomass accumulation [17]. The oil content in some species varies from 14 to 63% of the dry weight and the fatty acid composition includes C-14:0 to C-20:0 fatty acids [18, 19]. As lipid metabolism is induced to varying degrees by N starvation in various *Chlorella* species, this microalga is ideal for studying the mechanisms of lipid metabolism in algae. In a previous study, we defined four key stages of neutral lipid accumulation in *Chlorella sorokiniana* C3 after N starvation as the control stage (Cs), pre-oil droplet formation stage (PDFs), oil droplet formation stage (ODFs), and late-oil droplet formation stage (LDFs), and proposed a coupling mechanism of N starvation-induced neutral lipid accumulation and oxidative stress [20]. Notably, we suggested the possible role for cyclic electron flow (CEF) in supplying ATP for N starvation-induced lipid biosynthesis, which was found in turn to be regulated by the Ca^2+^ signal [21] through the calcium sensor protein (CAS) that regulates Pgrl1-mediated CEF [22], and proposed a Ca^2+^-regulated CEF that supplies ATP for N starvation-induced lipid biosynthesis in green alga [22]. Genetic engineering of the metabolic pathways of microalgae requires a comprehensive understanding of their regulation at the whole cell level, rather than at the single pathway level, including environmental stress-induced metabolic responses such as N starvation. Recent developments in high-throughput technologies have enabled the profiling of mRNA, proteins, and metabolites, giving rise to the fields of transcriptomics, proteomics, and metabolomics, respectively [23]. However, due to the lack of genome sequence information for most *Chlorella* strains, applications of transcriptomics and proteomics in *Chlorella* are limited. *Chlorella* has been shown to increase triglyceride production under stress conditions [2, 20–22, 24]; however, the mechanisms regulating oil production in microalgae are complex and poorly understood. Metabolite profiling provides an opportunity to study lipid metabolism in algae.

To identify the key contributors to lipid metabolic pathways in microalgae, we analyzed the metabolic profiles of three *Chlorella* strains that show significant differences in lipid biosynthesis at different stages of lipid accumulation. We found that N derived from amino acid catabolism was converted into certain amino acids and intermediate molecules. Excess C was then diverted into lipid metabolism to generate storage lipids, indicating that metabolism of N- and C-containing compounds contributes significantly to lipid metabolism in *Chlorella*. The findings of this study can be used to enhance lipid biosynthesis by genetically manipulating C/N metabolic pathways.

## Methods

### Strains

*Chlorella* strains *Chlorella sorokiniana* C1, *Chlorella* sp. C2, *Chlorella sorokiniana* C3, *Chlorella sorokiniana* C7, and *Chlorella* sp. A2 were collected from the wild; FACHB1, FACHB37, FACHB960, FACHB1068, FACHB1216, FACHB1222, FACHB1227, FACHB1552, FACHB1568, and FACHB1580 were obtained from the Freshwater Algae Culture Collection of the Institute of Hydrobiology (FACHB), Chinese Academy of Sciences.

*Chlamydomonas reinhardtii* knock-out mutants (Additional file 1: Table S1) deficient in glutamate synthase/NADH-dependent (NADH-GSN), glutamate synthase/Fd-dependent (Fd-GSN), glutamine synthetase (GS), aspartate aminotransferase (AST), alanine aminotransferase (ALT), pyruvate kinase (PK), and citrate synthase (CS) and their background strain CC4533 (cw15 mt-) were purchased from the *Chlamydomonas* Library Project (CLiP) (https://www.chlamylibrary.org/).

### Growth conditions and N− treatment

The N-sufficient medium (N+) used for *Chlorella* strains were full-strength BG11 medium [25]. The N-deficient medium (N−) was BG11 without NaNO_3_. *Chlorella* strains were cultured and subjected to N treatment as previously described [5, 20, 21]. *Chlorella* strains in the exponential phase were inoculated into a 1-l Erlenmeyer flask containing 500 ml BG11 medium at 25 °C with continuous illumination of 70 μmol m^−2^ s^−1^ and continuously bubbled with filtered air, and the initial OD_700_ is 0.05. For N− treatment, cells were harvested by centrifugation at 3000*g* for 3 min at 25 °C when they reached the midlogarithmic growth phase (OD_700_ approximately 0.8), and were then washed and resuspended in N− medium to OD_700_ 0.3.

The *C. reinhardtii* wild-type strain and *C. reinhardtii* knock-out mutant were grown as described by Tolleter et al. [26] with minor modifications. Cells in the exponential phase were inoculated into a 1-l Erlenmeyer flask containing 500 ml TAP medium at 25 °C with continuous illumination of 40 μmol m^−2^ s^−1^ and continuously bubbled with filtered air, and the initial OD_700_ is 0.05. The N− medium used for *C. reinhardtii* was TAP without NH_4_Cl. N− treatment of *C. reinhardtii* was the same as that for *Chlorella* strains.

### Algal growth analysis

The cell growth was monitored by OD_700_, where the samples were diluted to keep the OD_700_ between 0.2 and 0.8. Growth rate was calculated using Eq. (1), where *A*_1_ and *A*_2_ are defined as the OD_700_ at time 1 (*t*_1_) and time 2 (*t*_2_), respectively.

1 $${\text{Growth rate}} = \left( {A_{ 2} - A_{ 1} } \right)/\left( {t_{ 2} - t_{ 1} } \right)$$

### Nitrogen analysis

Cells were harvested by centrifugation and dried using a freeze dryer. After centrifugation, the residual total nitrogen concentration in the supernatant was detected using ion chromatography [27]. The total N contents in cells were determined using the Kjeldahl method according to Matejovic [28]. The protein content = total N contents in cells * 6.25.

### Lipid analysis

#### Thin-layer chromatography (TLC) analysis

A 10 ml culture at OD_700_ = 1 was harvested at 6000 *g* for 3 min, and the cell pellet was washed with fresh medium and centrifuged again. The harvested cell pellet was resuspended in 400 μl of methanol:chloroform mixture (1:1, v/v). The mixture was shaken for 2 min followed by phase separation using 120 μl of 1 M potassium chloride in 0.2 M phosphoric acid. Then the mixture was centrifuged at 12,000*g* at room temperature for 5 min, and the chloroform phase was transferred to a glass tube and dried under nitrogen. The residue was resuspended in a volume of 20 μl chloroform to get the lipid extracts. TLC analysis of lipid extracts from whole cells was performed according to Reiser and Somerville [29] with some modifications. TAGs were separated by developing the plates in hexane-ethyl ether (7.5:2.5, v/v). Samples were visualized by exposure to iodine vapor for approximately 10 min. 3 μl of each samples extracted at different time points was used for TLC analysis. Glyceryl trioleate (3 μl, 10 mg ml^−1^) was used as a reference substance for TAGs, and the neutral lipid content (mg ml^−1^ OD^−1^) was then determined accordingly using ImageJ (ver1.41, NIH) [30].

#### Confocal laser scanning microscopy (CLSM) analysis

Microscopy analysis of cells stained with Bodipy 505/515 (Sigma Aldrich, USA) was carried out using a confocal laser scanning microscope (Zeiss LSM 710 NLO). Non-fluorescent protoplast structures were visualized using the manufacturer’s recommended filter settings. Specific experimental processes were previously described [5, 20, 21]. A lipophilic fluorescent dye, Bodipy 505/515 (4,4-difluoro-1,3,5,7-tetramethyl-4-bora-3a, 4a-diaza-sindacene), was used to stain the intracellular oil-containing organelles, known as lipid bodies, with a final labeling concentration of 1 μM and 0.1% DMSO (v/v), according to Cooper et al. [31]. Bodipy fluorescence (green) was excited with an argon laser (488 nm) and detected at 505–515 nm. Autofluorescence (red) of algal chloroplasts was detected simultaneously at 650–700 nm.

#### Flow cytometry (FCM) analysis

Samples stained with Bodipy 505/515 were analyzed using a FACSAria flow cytometer (Becton–Dickinson, San Jose, CA, USA) equipped with a laser emitting at 488 nm and an optical filter FL1 (530/30 nm). The collected data were analyzed using FlowJo software (Tree Star, San Carlos, CA, USA).

### Experimental design and procedure of metabolome

To further characterize the metabolic response to N starvation in the three *Chlorella* strains, high-throughput technologies would be helpful. However, transcriptomics and proteomics approaches would be challenging, due to the lack of genome sequence information for the strains used in this study. Thus, metabolome analysis of global biochemical profiles were determined in samples of low (C3), medium (C1), and high (C2) lipid content at varying time points (0, 1, 2, and 6 day) with four biological replicates following nitrogen removal.

Following receipt, samples were inventoried and immediately stored at −80 °C. At the time of analysis, samples were extracted and prepared for analysis using Metabolon’s standard solvent extraction method. The extracted samples were split into equal parts for analysis on the GC/MS and UPLC-MS/MS platforms. Also included were three technical replicate samples created from a homogeneous pool containing a small amount of all study samples (“Client Matrix”). General platform methods are described in Additional file 2: Appendix A.

### Data quality and analysis: instrument and process variability

Instrument variability was determined by calculating the median relative standard deviation (RSD) for the internal standards that were added to each sample prior to injection into the mass spectrometers. Overall process variability was determined by calculating the median RSD for all endogenous metabolites (i.e., non-instrument standards) present in 100% of the Client Matrix samples, which are technical replicates of pooled client samples.

The present dataset comprises compounds of known identity (named biochemicals). Following log transformation and imputation of missing values (if any) with the minimum observed value for each compound, ANOVA contrasts were used to identify biochemicals that differed significantly between experimental groups. A summary of the numbers of biochemicals that achieved statistical significance (*p* ≤ 0.05), as well as those approaching significance (0.05 < *p* < 0.10), is shown in Additional file 1: Table S2 and Additional file 3. Analysis by two-way ANOVA identified biochemicals exhibiting significant interaction and main effects for experimental parameters of lipid content and time.

An estimate of the false discovery rate (*q* value) is calculated to take into account the multiple comparisons that normally occur in metabolomic-based studies. For example, when analyzing 200 compounds, we would expect to see about 10 compounds meeting the *p* ≤ 0.05 cut-off by random chance. The *q* value describes the false discovery rate; a low *q* value (*q* < 0.10) is an indication of high confidence in a result. While a higher *q* value indicates diminished confidence, it does not necessarily rule out the significance of a result. Other lines of evidence may be taken into consideration when determining whether a result merits further scrutiny. Such evidence may include (a) significance in another dimension of the study, (b) inclusion in a common pathway with a highly significant compound, or (c) residing in a similar functional biochemical family with other significant compounds. Refer to Additional file 2: Appendix B for general definitions and further descriptions of false discovery rate and other statistical tests used at Metabolon.

### Enzyme activity assays

Cells (10^7^ cells ml^−1^) were harvested by centrifugation at 3000*g* for 3 min, and the cell pellet was washed and then resuspended with 0.2 M sodium phosphate buffer (pH 7.8). The resuspended cells were homogenized at 4 °C and then centrifugated at 13,000*g* for 30 min at 4 °C. The supernatants were used for enzyme activity analysis directly. Protein content was assayed using BCA Protein Quantification Kit (TIANGEN, China). GS, AST, ALT, PK, and CS activities were measured using an GS Assay Kit, AST Assay Kit, ALT Activity Assay Kit, PK Activity Assay Kit, and CS Activity Assay Kit (Nanjing Bioengineering Institute, China), according to the manufacturer’s instructions.

NADH-GSN enzyme assay was performed as described by Lin and Kao [32] with minor modifications. 300 μl of reaction mixture contains 40 μl 50 mM l-glutamine, 50 μl 50 mM α-ketoglutarate, 20 μl 0.6 mM NADH, 30 μl enzyme sample, and 160 μl 50 mM PBS (pH 7.5). NADH-GSN activity was assayed at 25 °C after adding l-glutamine and determined by spectrophotometer at 340 nm. The decrease in absorbance was recorded for 5 min at 340 nm. NADH-GSN activity is defined as the rate of per micromoles NADH oxidation per minute per milligram of protein.

Fd-GSN enzyme assay was performed spectrophotometrically by following the glutamine-dependent oxidation of NADPH at 340 nm as described by Jamai et al. [33] and Misra and Oaks [34].

### Real-time RT-PCR analysis

Cells (10^7^ cells ml^−1^) were harvested and resuspended in a 1.5 ml micro-tube containing 1 ml TRIZOL Reagent (Invitrogen, USA). After precipitation in 100% isopropanol and washing in 75% ethanol, the RNA pellet was suspended in a suitable volume of DEPC water according to the manufacturer’s instructions. RNA solutions were quantified using a NanoDrop 3.0.0 (Coleman Technologies Inc., USA). Aliquots were stored at −70 °C.

The transcriptional expression of genes encoding NADH-GSN, Fd-GSN, GS, AST, ALT, PK, and CS was measured using real-time RT-PCR [35]. First-strand synthesis was carried out using a PrimeScript RT Reagent Kit With gDNA Eraser according to the manufacturer’s instructions (#RR047A, TAKARA). To perform the gene expression analyses, specific primer sets were designed to produce 100- to 200-bp PCR products (Additional file 1: Table S3). Quantitative real-time PCR was performed (three technical replicates on five biological replicates) using iTaq Universal SYBR Green Supermix (#172, Bio-Rad) and a Bio-Rad CFX96 Thermal Cycler (Bio-Rad, USA). Differences in expression were calculated according to the ‘delta–delta method’ [36], using CBLP as reference, which has been evaluated and confirmed by NormFinder algorithm [37].

### Statistical analyses

Each result shown is the mean of four or five biological replicates. Statistical analysis of the data was performed using the program SPSS-13 and significance was determined at 95 or 99% confidence intervals. *t* test was used to determine the means and SD of replicated studies. The significant differences between the control and test values were tested using one-way ANOVA test, and differences were considered to be significant at *p* < 0.05 or *p* < 0.01.

## Results

### Selection of three *Chlorella* strains with significant differences in lipid biosynthesis

We determined the lipid contents of 15 *Chlorella* strains (Additional file 4: Figure S1) and identified three *Chlorella* strains, i.e., *C. sorokiniana* (C1), *Chlorella* sp. (C2), and *C. sorokiniana* (C3), that have significant differences in lipid productivity and a high level of nucleotide sequence identity in their 18S rRNA gene sequences [38]. In addition, we found that although the growth of all three strains reached the lag phase at 12 days, significant differences (one-way ANOVA test, *p* < 0.05) in growth rates were evident (C3: 0.764 ± 0.044 > C1: 0.634 ± 0.032 > C2: 0.572 ± 0.023), which showed the similar trend with N consumption (C3 > C1 > C2) but contrary to lipid contents (C3 < C1 < C2) (Additional file 4: Figure S2A, B, C and Figure S3). Thus, we analyzed the metabolites in these three strains, with the aim of identifying the key regulatory pathways underlying lipid synthesis.

**Fig. 1:**
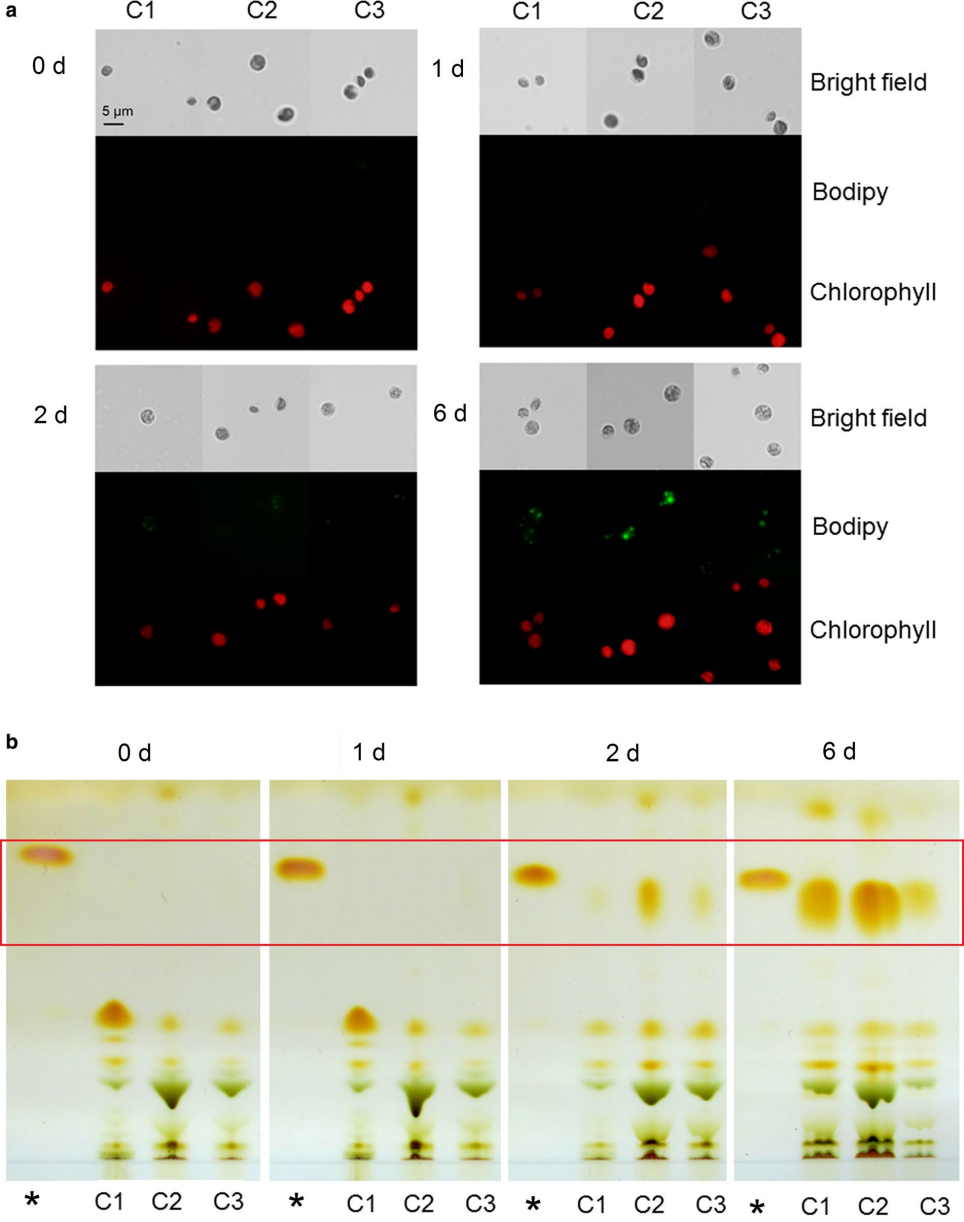
Lipid accumulation of three *Chlorella* strains with different lipid contents grown under nitrogen starvation. Lipid accumulation analyzed by CLSM (**a**) and TLC (**b**) after 0, 1, 2, and 6 day of N starvation. CLSM (**a**) images of cells with Bodipy 505/515 fluorescence (*green*) and Chl autofluorescence (*red*) in each treatment were recorded. The size of the *scale bar* is shown directly on the image. *Asterisk* symbol in **b**, glyceryl trioleate as loading standard. The expected lipid bands for further clarity were marked using *red box*. All of the *figures* are representative of three replicated studies with similar findings

In our previous study, we defined four key stages of neutral lipid accumulation in *C. sorokiniana* (C3): 0, 0–0.5, 0.5–2, and 2–8 day after N starvation [20]. Although more lipids accumulated in *C. sorokiniana* (C1) and *Chlorella* sp. (C2) than in *C. sorokiniana* (C3), the overall trends of neutral lipid accumulation in all three strains were similar, exhibiting similar neutral lipid accumulation stages (Additional file 4: Figure S3). Lipids accumulate extensively in *Chlorella* cells during N starvation. We thus examined the contributions of the metabolism of N- and C-containing compounds to lipid biosynthesis in the selected strains after 0, 1, 2, or 6 day of N starvation. To compare neutral lipid accumulation in the strains, we stained the cells with Bodipy 505/515, a fluorescent marker that stains intracellular lipids, and examined the cells using confocal laser scanning microscopy (CLSM). Although enhanced green fluorescence signals from Bodipy 505/515 were detected as time progressed in all three *Chlorella* strains, after 6 day of N starvation the strongest green fluorescence signal was observed in C2 and the weakest green fluorescence signal was detected in C3 (Fig. 1a, 6 day).

We also examined the intracellular lipid levels of the three strains at various stages of N starvation using TLC. In accordance with the CLSM results (Fig. 1a), we observed significant lipid accumulation after 6 days of N starvation (Fig. 1b, 6 day; Additional file 4: Figure S4) and defined C2, C1, and C3 as having high, medium, and low lipid contents, respectively. We then subjected these *Chlorella* strains to metabolic tests to pinpoint differences in the key stages of lipid biosynthesis between the strains.

### Nitrogen assimilation for amino acid metabolism is important for lipid metabolism

**Fig. 2:**
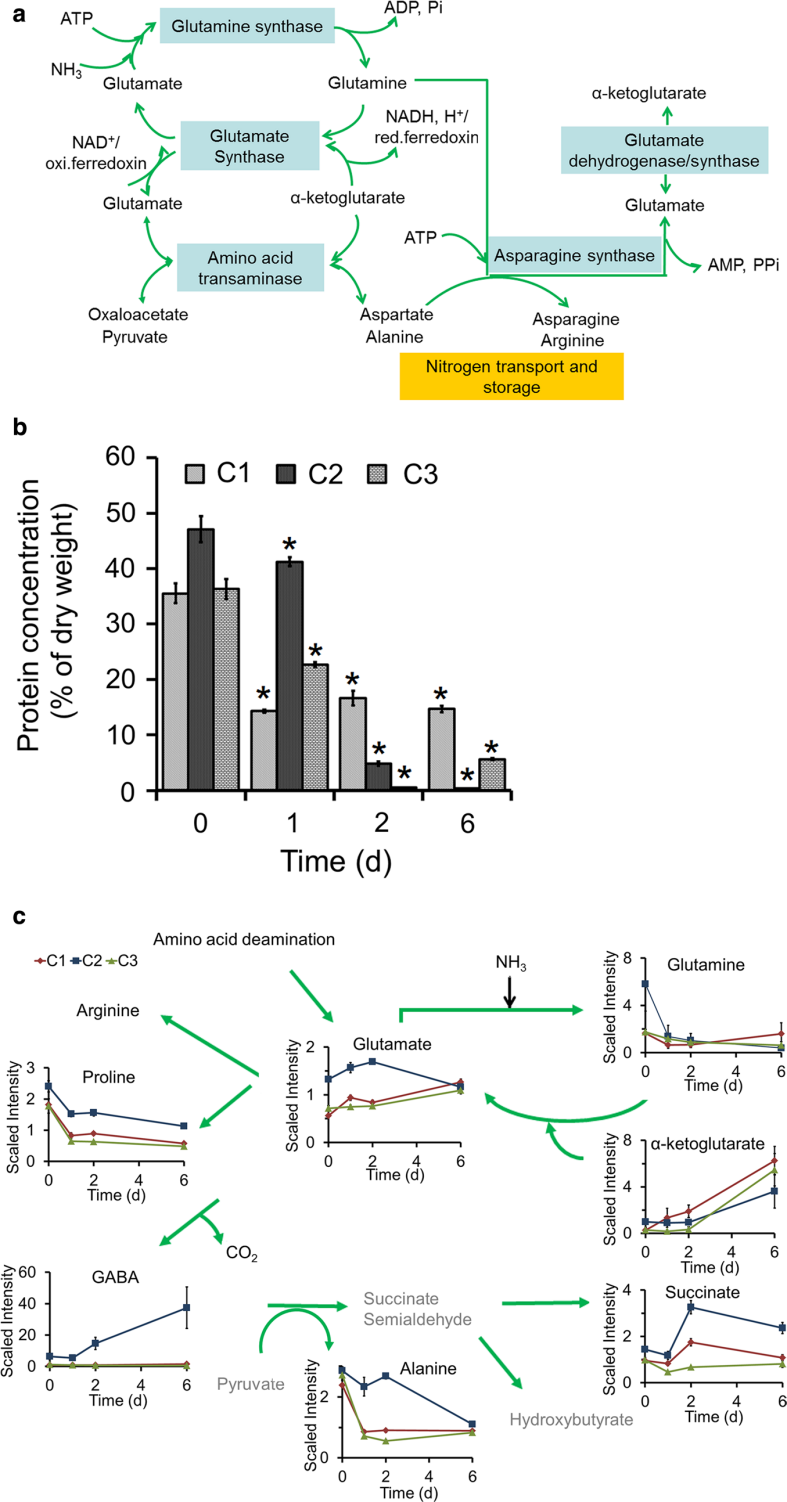
Main nitrogen assimilation related to amino acid metabolism pathway in three *Chlorella* strains. **a** glutamate–glutamine system and corresponding transaminase pathways; **b** protein concentration in three *Chlorella* strains under N− treatment, all data points in the current and following *figures* represent the means and SD of five biological replicates (*t* test, *p* < 0.05), and the significance of the differences between the 0 day of each strain and other test values in 1, 2, and 6 day in the same strain was tested using a one-way ANOVA. *p < 0.05; **c** nitrogen assimilation and re-distribution from glutamate. The line plot *Y-axis* represents median-scaled (ion counts under the curve) intensity data. The intensity data have no units. In all plots, “1.0” represents the median of all the sample values detected for that compound. After determination of the median, any null values are imputed (substituted) with the minimum detected value for that compound

Metabolome analysis indicated that *Chlorella* strains produce 220 biochemical compounds (Additional file 4: Figure S5), including 59 amino acids, 46 carbohydrates, 64 lipids, 14 cofactors, prosthetic groups, and electron carriers, 21 nucleotides, 16 peptides, and 1 xenobiotic. N assimilation in bacteria, photosynthetic algae, and higher plants occurs via the glutamine synthetase and glutamate synthase pathways [39–41]. Once N is assimilated into glutamate and glutamine, amino-transferases re-distribute it to other molecules, including amino acids (Fig. 2a), which are essential for protein synthesis. When N is insufficient, the cell protein content decreased significantly (one-way ANOVA test, *p* < 0.05) and N was possibly recycled into amino acids to mitigate stress (Fig. 2b). In strain C2, glutamine levels decreased fourfold between 0 and 1 day of N starvation and progressively decreased thereafter (Fig. 2c). In strains C3 and C1, glutamine levels remained unchanged relative to 0 day throughout the treatment (Fig. 2c). Glutamate, the product of N assimilation, increased gradually in C3 and C1. In C2, glutamate levels increased during the first 2 day and then declined, along with a dramatic increase in gamma-aminobutyrate (GABA) (Fig. 2c). During the early stages of culture under N starvation, increases in glutamate may reflect catabolism of amino acids in C2, which may be converted to GABA by a decarboxylase reaction, leading to N storage in the form of GABA (Fig. 2c). The levels of some amino acids, including glutamate, proline, GABA, alanine, and succinate, in C2 were much higher than in C1 and C3 at all time points, suggesting that N assimilation and incorporation into these amino acids and relevant metabolic pathways make an important contribution to lipid biosynthesis. Furthermore, the levels of succinate in the three strains corresponded to the levels of lipids, indicating that N assimilation and incorporation via the succinate pathway (e.g., GABA to succinate and the TCA cycle) play important roles in lipid metabolism.

**Fig. 3:**
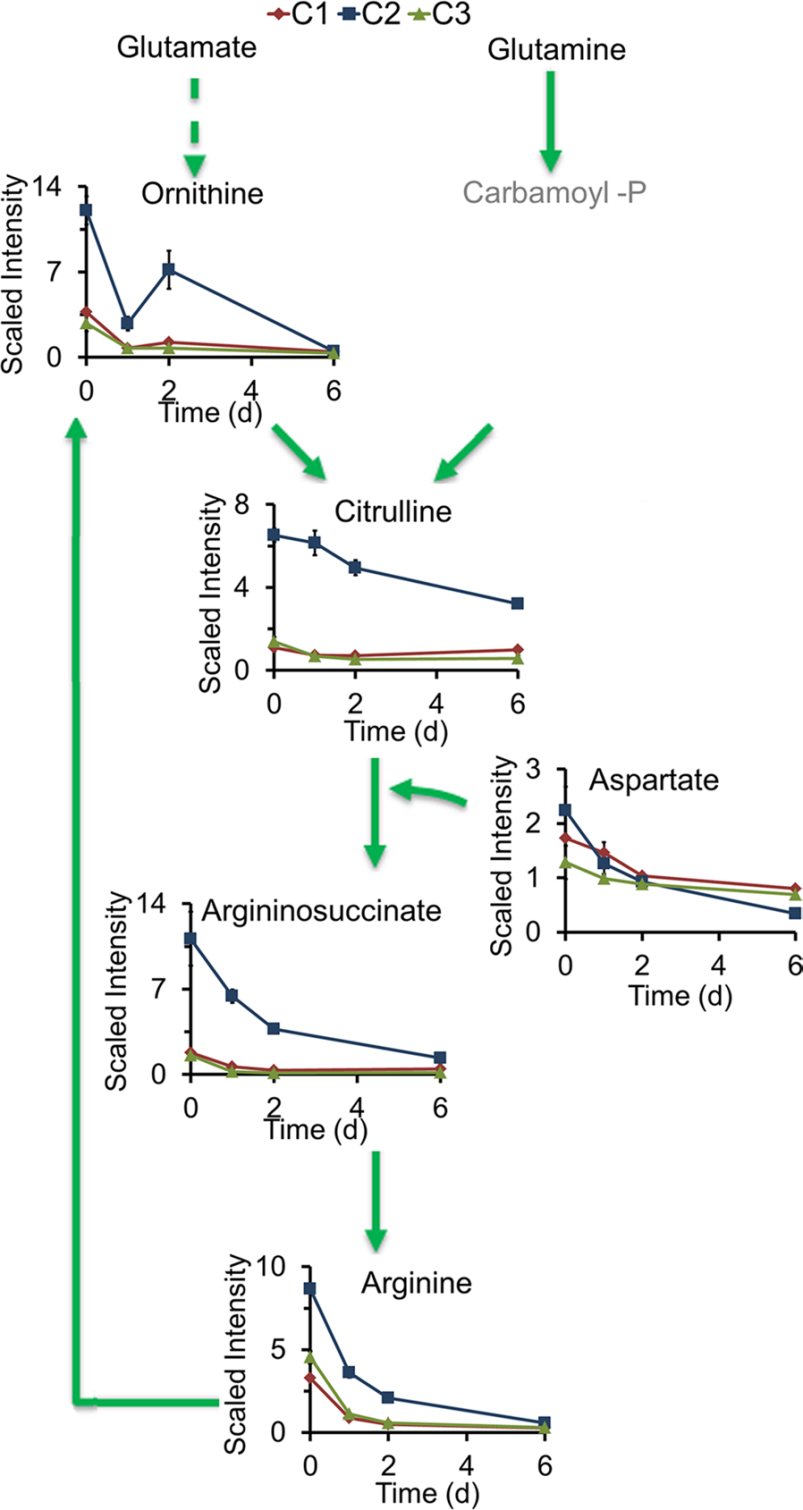
Arginine biosynthesis process in three *Chlorella* strains. The compound names with *gray* were undetected compounds. *Solid arrow* one step of metabolic flow, *dotted arrow* more than one step of metabolic flow. The line plot *Y-axis* represents median-scaled (ion counts under the curve) intensity data. The intensity data have no units. In all plots, “1.0” represents the median of all the sample values detected for that compound. After determination of the median, any null values are imputed (substituted) with the minimum detected value for that compound

In the arginine biosynthesis pathway (Fig. 3), the levels of all metabolites were much higher in C2 than in C1 and C3. For example, after 2 day of N starvation, argininosuccinate levels were about 32-fold higher in C2 than in C3 (Fig. 3), suggesting that the arginine metabolic pathway is also important in lipid biosynthesis. Amino acid levels were generally higher in C2 than in C3, and to some extent in C1 (Figs. 2, 3; Additional file 3), indicating that N assimilation related to amino acid metabolism is important in lipid metabolism.

In strain C2, glutamine, asparagine, and arginine (involved in N storage and transport) decreased precipitously between 0 and 1 day of N starvation and continued to decrease as the treatment progressed (Figs. 2, 3; Additional file 3). Most other amino acids, except for aspartate, lysine, and proline, increased between 0 and 1 day and then deceased rapidly by 6 day of treatment. The initial increase in amino acids was most likely due to N salvage from protein degradation. Molecules derived from post-translational modification, such as trans-4-hydroxyproline, methionine sulfoxide, and N-acetyl-amino acids, as well as amino acid degradation products, such as β-hydroxyisovalerate, 4-guanidinobutanoate, pipecolate 2-aminoadipate, and 2-aminobutyrate, showed a similar pattern of change over time (shown in Additional file 3). These results imply that in response to N starvation, major N salvage in strain C2 may result from the catabolism of proteins and non-essential amino acids, with the resulting N being diverted to essential metabolic pathways, such as the TCA cycle, which is important for lipid biosynthesis.

### Nitrogen assimilation for nucleic acid metabolism is not the major pathway contributed to lipid metabolism

**Fig. 4:**
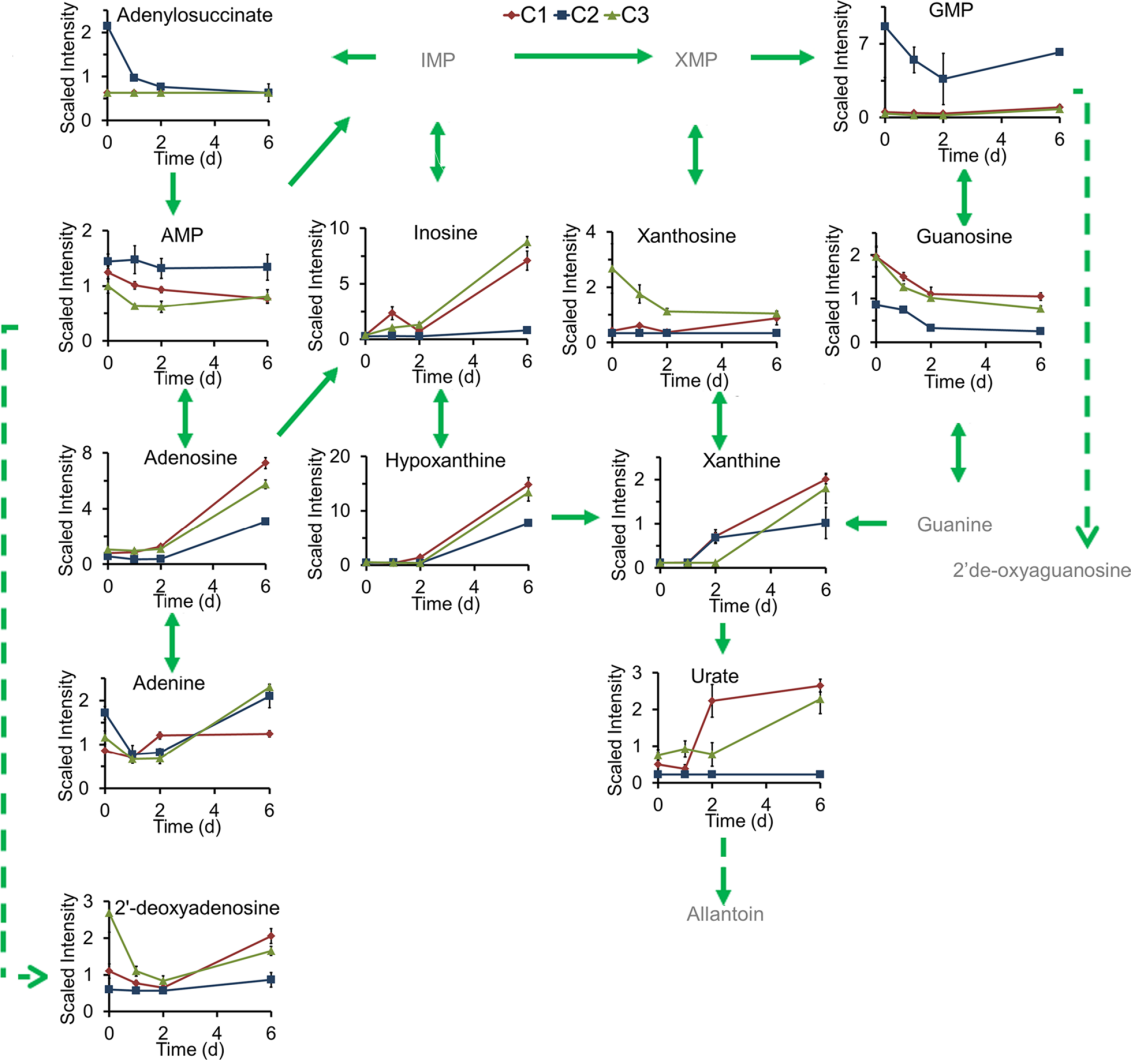
Main nitrogen assimilation related to purine metabolism process in three *Chlorella* strains. The compound names with *gray* were undetected compounds. *Solid arrow* one step of metabolic flow, *dotted arrow* more than one step of metabolic flow. The line plot *Y-axis* represents median-scaled (ion counts under the curve) intensity data. The intensity data have no units. In all plots, “1.0” represents the median of all the sample values detected for that compound. After determination of the median, any null values are imputed (substituted) with the minimum detected value for that compound

**Fig. 5:**
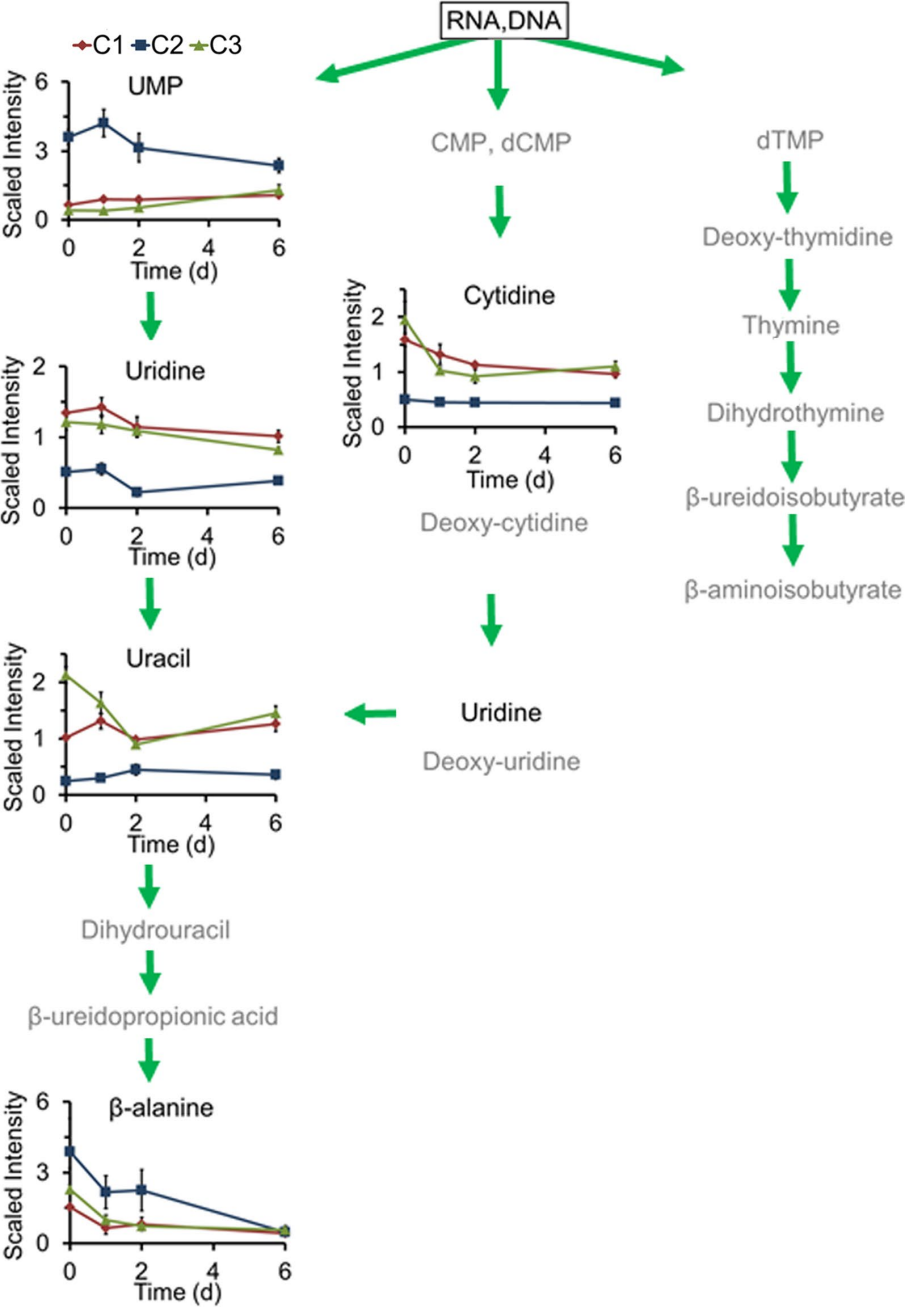
Main nitrogen assimilation related to pyrimidine metabolism process in three *Chlorella* strains. The compound names with *gray* were undetected compounds. The line plot *Y-axis* represents median-scaled (ion counts under the curve) intensity data. The intensity data have no units. In all plots, “1.0” represents the median of all the sample values detected for that compound. After determination of the median, any null values are imputed (substituted) with the minimum detected value for that compound

Although AMP, GMP, and an intermediate in purine biosynthesis, adenylosuccinate, were present at higher levels in C2 than in C3 and C1 (most likely due to nucleic acid turnover) (Fig. 4), the products of purine degradation (urate, xanthine, adenosine, deoxyadenosine, inosine, and xanthosine) were present at higher levels in C3 and C1 than in C2 (Fig. 4). For the pyrimidine metabolic pathway, the levels of UMP and the end product β-alanine were higher in C2 than in C3 and C1, but the degradation products of pyrimidine, including cytidine, uridine, and uracil, were present at higher levels in C3 and C1 (Fig. 5). These results imply that nucleic acid catabolism plays a greater role in N salvage in strains C3 and C1 than in strain C2, in which N may be salvaged via amino acid catabolism. However, as the lipid accumulation in C2 was greater than in C1 and C3 (Additional file 4: Figure S4, one-way ANOVA test, *p* < 0.05), the N assimilation and distribution pathways related to nucleic acid metabolism are not the major pathway contributed to the lipid metabolic pathway.

### Glutamine synthetase and glutamate synthase pathways mediate nitrogen assimilation

**Fig. 6:**
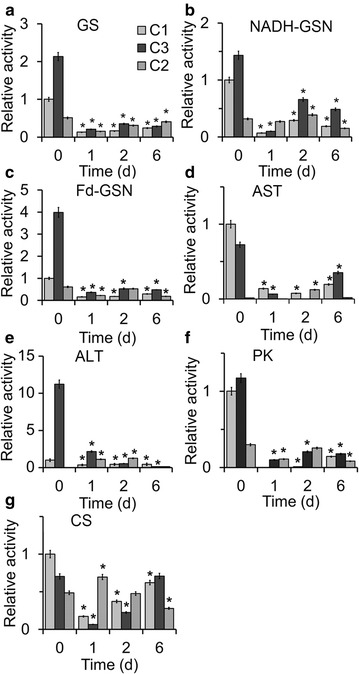
The relative enzyme activities in three *Chlorella* strains. The relative enzyme activities of glutamine synthetase (GS) (**a**), glutamate synthase/NADH-dependent (NADH-GSN) (**b**), glutamate synthase/Fd-dependent (Fd-GSN) (**c**), aspartate aminotransferase (AST) (**d**), alanine aminotransferase (ALT) (**e**), pyruvate kinase (PK) (**f**), and citrate synthase (CS) (**g**) were detected after 0, 1, 2, and 6 day of N starvation, and control (0 day of C1) value of each enzyme activity was set to 1 for easy comparison. The significance of the differences between the 0 day of each strain and other test values in 1, 2, and 6 day in the same strain in each *panel* was tested using a one-way ANOVA. *p < 0.05

To further investigate the contribution of N assimilation to lipid metabolism, we measured the activity levels of several key enzymes involved in N assimilation pathways, including NADH-GSN, Fd-GSN, GS, AST, and ALT. In the catalysis of NADH-GSN, Fd-GSN, and GS, extracellular and/or intracellular N sources are fixed via the glutamate–glutamine cycle, followed by re-distribution of the assimilated N by AST and ALT into other key molecules, including amino acids and nucleic acids. The activities of all five enzymes in the three strains initially decreased, but then increased during the N starvation treatment (Fig. 6), indicating that the strains were adapting to N starvation and that short-term N salvage from the catabolism of amino acids or nucleic acids maintained basic cell functions, including the regulation of lipid metabolism. In the initial stage of treatment (0–1 day) in C3 and C1, the activities of all of the enzymes decreased significantly (one-way ANOVA test, *p* < 0.05), but the activities in C2 decreased slightly (Fig. 6), suggesting that N assimilation involved in N salvage from the catabolism of amino acids via this system played important regulatory roles in lipid metabolism.

### Metabolism of energy storage molecules and carbon distribution for lipid metabolism

**Fig. 7:**
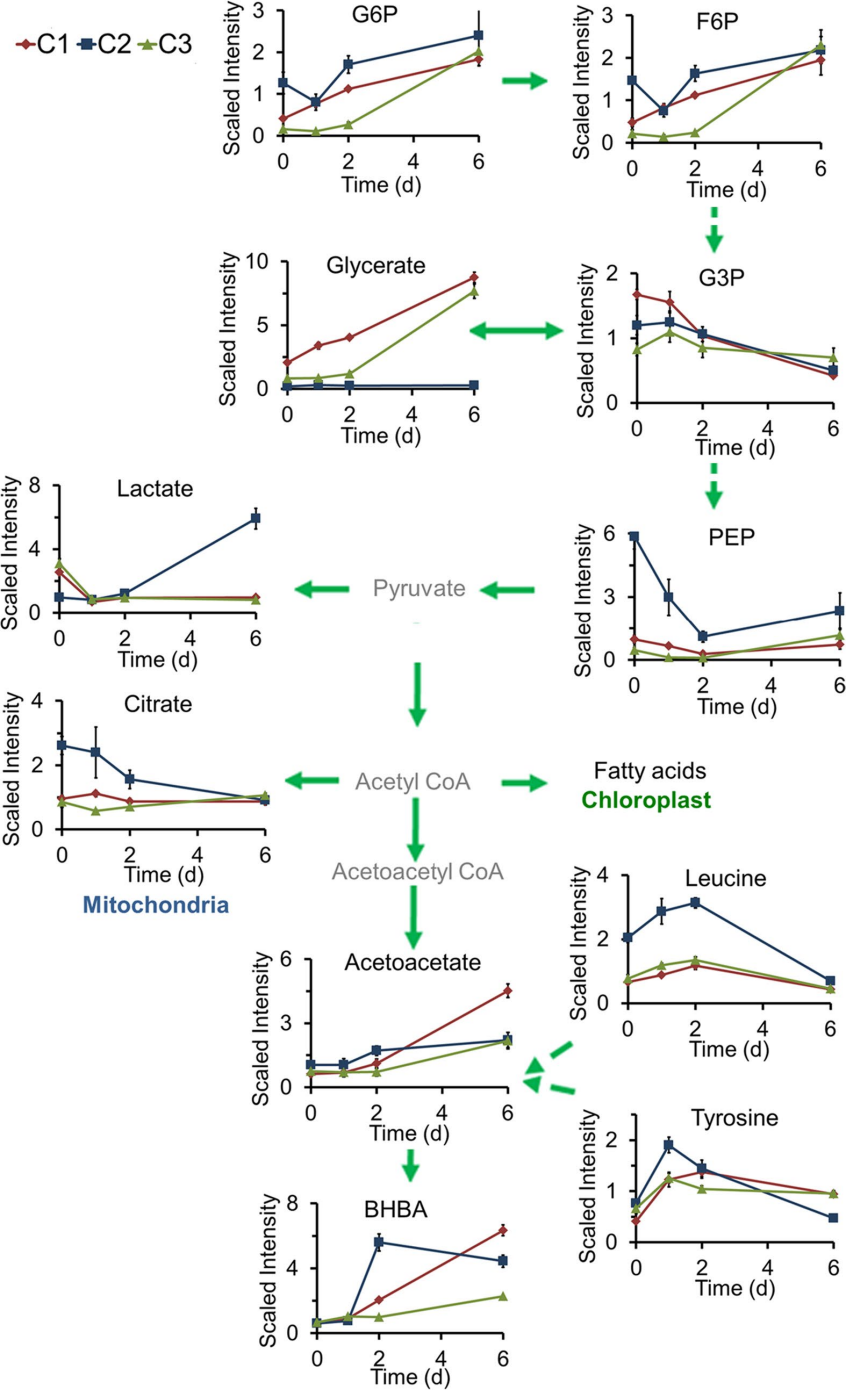
Main carbon sink and distribution from glycolysis process in three *Chlorella* strains. The compound names with *gray* were undetected compounds. *Solid arrow* one step of metabolic flow, *dotted arrow* more than one step of metabolic flow. The line plot *Y-axis* represents median-scaled (ion counts under the curve) intensity data. The intensity data have no units. In all plots, “1.0” represents the median of all the sample values detected for that compound. After determination of the median, any null values are imputed (substituted) with the minimum detected value for that compound

Carbon metabolic pathways provide structural components, reducing power, and energy for the assimilation and utilization of N in photosynthetic organisms. Therefore, these processes are closely integrated and regulated to maintain the C/N balance [42, 43]. We observed several interesting differences in carbon metabolic pathways between the three strains. The levels of glucose-6-phosphate (G6P) (which may be obtained from photosynthesis or glycolysis), phosphoenolpyruvate (PEP), and leucine were higher in C2 than in C3 and C1 at all stages of N starvation treatment (Fig. 7), indicating that these upstream intermediate products of glycolysis influence lipid metabolism. Concurrently, lactate, which was initially lower in C2 than in the other two strains, progressively increased in C2 to a level that was sixfold higher than the level at 0 day. Further, there was a progressive increase in 3-hydroxybutyrate (BHBA) in all three strains (Fig. 7). Lactate is produced from pyruvate in the cytosol during hypoxia. It is possible that, under N starvation conditions, strain C2 becomes hypoxic and C is diverted predominantly into BHBA in strains C1 and C3 and into lactate and BHBA in strain C2, and that all of these C-containing compounds play roles in distributing C for lipid and carbohydrate metabolism. Many sugar molecules, including glucose, fructose, galactose, raffinose, glucoheptose, maltotetraose, mannose, ribose, ribulose, arabinose, and N-acetylglucosamine, were present at higher levels in C3 and C1 than in C2 at the various stages of treatment (shown in Additional file 3), signifying that C was diverted into carbohydrate storage molecules in C3 and C1. In addition, the decrease in citrate in strain C2 indicated that C flow from the TCA cycle was diverted to lipid metabolism (Fig. 7).

Two key enzymes in the carbon metabolic pathway, PK and CS, were also detected in the strains during N starvation treatment. The activity of PK, which catalyzes the transformation between pyruvate and PEP, initially decreased and then increased (Fig. 6), indicating that the cells adapted to N deficiency to maintain C assimilation for energy storage. In addition, the activity of CS, which catalyzes the transformation between acetyl CoA and citrate, initially decreased in strains C1 and C3, but then increased progressively thereafter. The opposite trend was observed in C2. This suggests that the assimilated C in C1 and C3 was diverted to the TCA cycle to produce ATP and carbohydrates, but was redirected from the TCA cycle to lipid metabolism in C2.

### The contribution of nitrogen/carbon metabolism to lipid metabolism in oil-rich algae

Besides the physiological changes and metabolites differentia, the changes in transcription level related to nitrogen/carbon metabolism are more conducive to clarify the contribution of nitrogen/carbon metabolism to lipid metabolism. However, due to the lack of genome sequence information and the difficulty of molecular manipulation for most *Chlorella* strains, it was difficult to perform qPCR analysis and obtain the knock-out mutants of key enzymes in this study. To further confirm the contributions of nitrogen/carbon metabolism in oil-rich algae to lipid metabolism, the conclusion from *Chlorella* strain C2, we used model green alga *Chlamydomonas* with high lipid production.

**Fig. 8:**
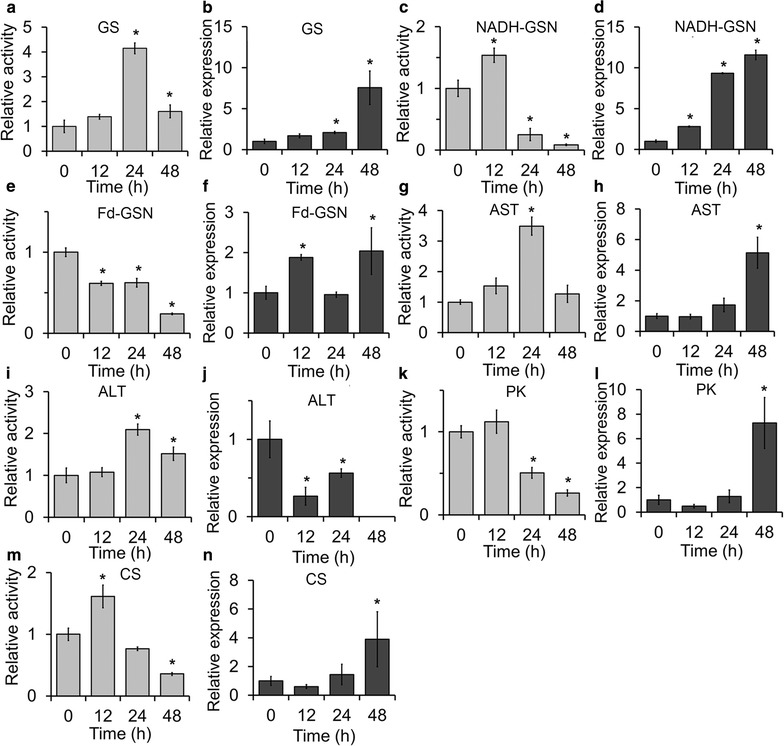
The relative enzyme activities and gene transcript levels in *Chlamydomonas reinhardtii* strain CC4533. The relative enzyme activities and gene transcript levels of glutamine synthetase (GS) (**a**, **b**), glutamate synthase/NADH-dependent (NADH-GSN) (**c**, **d**), glutamate synthase/Fd-dependent (Fd-GSN) (**e**, **f**), aspartate aminotransferase (AST) (**g**, **h**), alanine aminotransferase (ALT) (**i**, **j**), pyruvate kinase (PK) (**k**, **l**), and citrate synthase (CS) (**m**, **n**) were detected after 0, 12, 24, and 48 h of N starvation, and control (0 h) value of each enzyme activity or gene transcript level was set to 1 for easy comparison. The significance of the differences between the 0 h and other test values in each *panel* was tested using a one-way ANOVA. *p < 0.05

We monitored changes in enzyme activity and the corresponding transcript levels of NADH-GSN, Fd-GSN, GS, AST, ALT, PK, and CS in *C. reinhardtii* strain CC4533. Because the N starvation-mediated induction of oil formation in *C. reinhardtii* is much faster than in *Chlorella*, we set induction times of 12, 24, and 48 h. After N starvation, the activities of several key enzymes in N assimilation, including NADH-GSN (Fig. 8c), GS (Fig. 8a), AST (Fig. 8g), and ALT (Fig. 8i), initially increased (12–24 h), which further demonstrated that N salvaging was occurring. We next analyzed the transcript levels of the genes encoding five enzymes (NADH-GSN, Fd-GSN, GS, AST, and ALT) involved in N assimilation in *C. reinhardtii* CC4533 using real-time fluorescence quantitative PCR and found that the transcripts of all these genes except ALT increased (Fig. 8), thus increasing the activity of their corresponding enzymes after 12–24 h of N starvation, confirming that enzyme activities increased in response to N starvation. As these enzymes enabled the cell to withstand N starvation, the transcripts were elevated significantly (one-way ANOVA test, *p* < 0.05) to mitigate the drastic decline of enzyme activities at 48 h. Although the enzyme activity of ALT increased, the level of the corresponding gene transcript decreased, indicating that the activity of this enzyme is not regulated at the transcriptional level, but possibly at the translational level. Similarly, the activities of PK and CS, which both regulate carbon catabolism and the re-distribution of C via glycolysis and the TCA cycle to lipids or other energy storage molecules, increased during the first 12 h of N starvation, and the levels of the corresponding gene transcript also increased (Fig. 8).

**Fig. 9:**
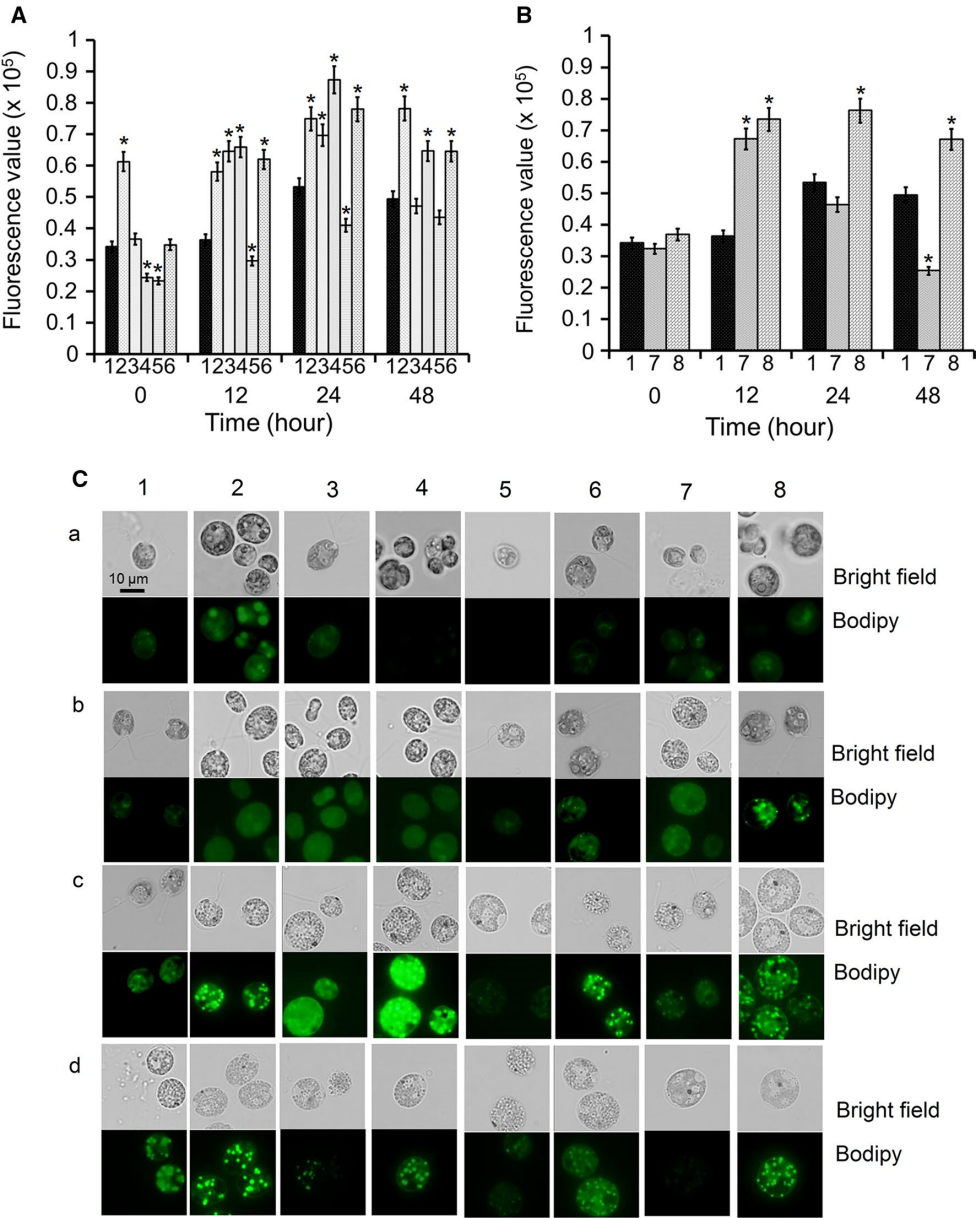
Lipid accumulation of *Chlamydomonas reinhardtii* strain CC4533 and its knock-out mutants. Lipid accumulation of *Chlamydomonas reinhardtii* strain CC4533 (*1*) and its knock-out mutants deficient in glutamate synthase/NADH-dependent (*2*), aspartate aminotransferase (*3*), glutamine synthetase (*4*), alanine aminotransferase (*5*), glutamate synthase/Fd-dependent (6), pyruvate kinase (*7*), and citrate synthase (*8*) cells labeled in vivo with Bodipy 505/515 were analyzed by FCM (**A**, **B**) and CLSM (**C**) after 0 h (*a*), 12 h (*b*), 24 h (*c*), and 48 h (*d*) of N starvation. **A**, **B** The control (0 h of sample 1) value of lipid content was set to 1 for easy comparison; the significance of the differences between the CC4533 (*1*) and other mutants in each time point was tested using a one-way ANOVA. *p < 0.05. **C** CLSM images of cells with Bodipy 505/515 fluorescence (*green*) in each treatment were recorded, all of the *figures* are representative of three replicated studies with similar findings, and the size of the *scale bar* is shown directly on the image

Using the *C. reinhardtii* knock-out mutants deficient in NADH-GSN, Fd-GSN, GS, AST, ALT, PK, or CS in the CC4533 background (Additional file 4: Figure S6), we monitored neutral lipid synthesis using both FCM and fluorescence microscopy. Compared with the background strain, lipid metabolism in most of the knock-out mutants was significantly enhanced (one-way ANOVA test, *p* < 0.05) (Fig. 9), supporting the contributions of N/C assimilation and distribution via these pathways to lipid metabolism. However, neutral lipid synthesis was blocked by a deficiency of PK. These results suggested that interfering with N assimilation or carbon metabolism via genetic manipulation may alter lipid biosynthesis in microalgae. However, due to the complexity of metabolic pathways in algal cells, a deficiency of an individual enzyme (e.g., ALT) would likely have little effect on lipid metabolism.

## Discussion

Recent developments in high-throughput technologies have enabled the profiling of metabolites, including N-containing metabolites, giving rise to the field of metabolomics [23, 44]. Machado et al. [45] performed a metabolic analysis on freshwater *Nitzschia palea* strains, BR006 and BR022, collected from a eutrophic pond and from an artificial rock, respectively, and demonstrated that these strains developed distinct metabolic responses to different N conditions. BR022 maintained cellular homeostasis and slowed down growth in response to N availability, whereas BR006 maximized its growth rate even under N limitation by triggering a stress response that relocated carbon to lipid compounds and arrested growth after N exhaustion. A combination of physiological, transcriptional, and metabolic approaches revealed molecular and metabolic modifications that occur when the marine diatom *Phaeodactylum tricornutum* is grown under N-deprived conditions. These modifications indicated that *P. tricornutum* responded to N deprivation through increasing N transport and assimilation and utilizing organic N resources [16]. Following exposure to N deprivation, *P. tricornutum* reduced the biosynthesis of N-containing compounds and increased the recycling of N compounds such as amino acids, proteins, and nucleic acids. Similarly, a closely related symbiotic *Desmodesmus* sp. isolated from a hydroid was found to degrade Rubisco in response to N starvation, presumably to salvage the N contained in this protein [46]. In a previous study, we found that N is salvaged from nucleotides in *Chlorella* C3 grown under N starvation conditions, which enhances the expression of the obligatory response proteins (e.g., CaM and CAS), and thereby increase N-use efficiency [22]. In this study, we found that the total protein level and the amino acids (proline, GABA, alanine, arginine, and succinate) involved in N assimilation and re-distribution in C2 were higher than in the other two strains before N− treatment (time point 0), suggesting the surplus proteins and amino acids possibly provide substrates for N salvage and re-distribution under N− conditions, and the significant differences between C2 and C1 and C3 at time point 0 might be also an important reason for the differences in lipid metabolism significance. In a study, Gressler et al. [47] investigated the biochemical composition of four species of marine benthic algae (*Laurencia filiformis*, *L. intricata*, *Gracilaria domingensis,* and *G. birdiae*), and also found that the highest lipid content was detected in the algae with the highest protein level and amino acid level. Through metabolic profiling analysis, physiological tests, and real-time RT-PCR analysis (Figs. 2, 3, 4, 5, 6 and 8), we further showed that N salvage and re-distribution from proteins and amino acid catabolism via GS/GSN and the corresponding transaminase pathways in the C2 strain represent an important metabolic response of microalgae that contributes to lipid metabolism. In addition, in inverse proportion to its lipid-producing capacity, the levels of phospholipids, which are involved in membrane remodeling during growth and development [48], was lower in C2 than in C1 and C3 (shown in Additional file 3), implying that the high oil producing strain has a lower growth rate following re-suspension in N− medium (Additional file 4: Figure S2D, Figure S3).

As N assimilation requires energy, reducing molecules, and carbon skeletons, this process is influenced by C metabolic networks involved in photosynthesis, day respiration, and photorespiration. Park et al. [49] analyzed metabolic changes in *C. reinhardtii* grown under N deprivation and found that a N-sensing system was transduced to C- and N-responsive pathways, leading to the down-regulation of C assimilation and chlorophyll biosynthesis and an increase in N metabolism and lipid biosynthesis. The authors found that the expression of nearly all enzymes that catalyze N assimilation increased significantly. Our study as well as previous studies [20–22] showed that a number of key biological functions, including photosynthesis and C metabolism, were either up- or down-regulated under N-limited conditions. Glutamate, which plays a key role in the transamination step in the catabolism and biosynthesis of many amino acids, may also be converted to GABA (Fig. 2). Under N-limiting conditions, this is the preferred pathway for the partitioning of glutamate. α-ketoglutarate provided the C backbone for N assimilation. The C in GABA is diverted to the TCA cycle as succinate with the concurrent transfer of the amine residue to pyruvate to form alanine. These N/C distribution pathways mentioned above (e.g., GABA to succinate and the TCA cycle) play important roles in lipid metabolism. Glycolysis and the TCA cycle are associated with energy metabolism and provide C skeletons for lipid biosynthesis (Fig. 2). Guerra et al. [50] analyzed metabolite and transcript levels of central carbon metabolic pathways and determined the average fluxes and quantum requirements for the synthesis of proteins, carbohydrates, and fatty acids in the diatom *P. tricornutum*. They revealed that the GS/GSN pathway is the main regulation site for the nitrate-dependent control of C partitioning between protein and lipid biosynthesis, while the α-ketoglutarate/glutamate/glutamine metabolite ratio serves as a transcriptional signal, possibly related to the redox poise of intermediates in the photosynthetic electron transport system. In this study, when N is insufficient, the protein level decreased (Fig. 2b), and excess C was re-distributed between carbohydrates and lipid biosynthesis. Regulated via GS/GSN pathway, C was diverted into lipids in C2 but carbohydrate storage molecules in C3 and C1 (shown in Additional file 3). Moreover, for further confirming the contribution of nitrogen/carbon metabolism to lipid metabolism, the changes in transcription level related to nitrogen metabolism were detected in the model green alga *Chlamydomonas* with high lipid production. To withstand N starvation and mitigate the drastic drop in protein abundance, the transcription of enzymes involved in GS/GSN and the corresponding transaminase pathways was elevated. After extended periods of N starvation, mRNA levels rising while total enzymatic activity (which reflects protein abundance) declined (Fig. 8).

The limited understanding of the biology of microalgae is a bottleneck for further developing microalgal biofuels [4]. However, advances in sequencing and genome technologies and post-genomics, proteomics, and metabolomics approaches [51] will reveal which regulatory genes in photosynthetic microalgae (e.g., *C. reinhardtii*) can be manipulated to increase lipid production. Gargouri et al. [52] used a combined omics (transcriptomics, proteomics, and metabolomics) approach to identify several regulatory hubs that control various aspects of cellular metabolism, including N assimilation, central metabolism, photosynthesis, and lipid metabolism, in *C. reinhardtii* cells shifted from N-replete to N-depleted conditions. As there are neither genome information nor molecular manipulation protocols available in *Chlorella*, our study, based on the analysis of model green alga *C. reinhardtii* strain CC4533 and its knock-out mutants deficient in enzymes involved in N assimilation and C metabolism, confirmed that these enzymes contribute to lipid metabolism. Our results indicate that the metabolites and enzymes involved in N assimilation and C metabolism regulate lipid metabolism. Thus, enhancing or restricting the N assimilation pathway or C metabolism via genetic manipulation may increase lipid biosynthesis in microalgae.

Alipanah et al. [16] found that C metabolism was restructured through down-regulation of the Calvin cycle and the coordinated up-regulation of glycolysis, the TCA cycle, and pyruvate metabolism, leading to the funneling of C sources into lipid metabolism. Our metabolic profiling analysis showed that some metabolic pathways related to N/C assimilation and distribution make important contributions to lipid metabolism. It is suggested that not only carbon but also nitrogen assimilation and distribution pathways contribute to lipid biosynthesis. In regular culture condition (N+), N is assimilated into glutamate and glutamine, and then re-distributed to other molecules via amino-transferases system for synthesis of protein or other nitrogen-containing compounds. When N is insufficient, nitrogen-containing compounds especially cell protein content decreased significantly. In response to N starvation, major N salvage from the catabolism of proteins and non-essential amino acids was stored as some essential amino acids and intermediate molecules (particularly proline, alanine, arginine, succinate, and gamma-aminobutyrate) via the corresponding metabolic pathways, such as the glutamate–glutamine pathway, the corresponding transaminase pathway, GABA pathway, and TCA cycle, which led to carbon–nitrogen disequilibrium and made important contributions to lipid metabolism. In addition, nucleic acid catabolism and corresponding distribution pathways also play a role in N salvage when N is insufficient, but it is not the major pathway contributed to the lipid metabolic pathway. Following carbon–nitrogen disequilibrium, excess carbon obtained from photosynthesis or glycolysis was re-distributed into carbon-containing compounds, such as G6P, F6P, PEP, lactate, citrate, BHBA, and leucine, and then diverted into lipid metabolism via the GABA pathway, glycolysis, and the TCA cycle.

**Fig. 10:**
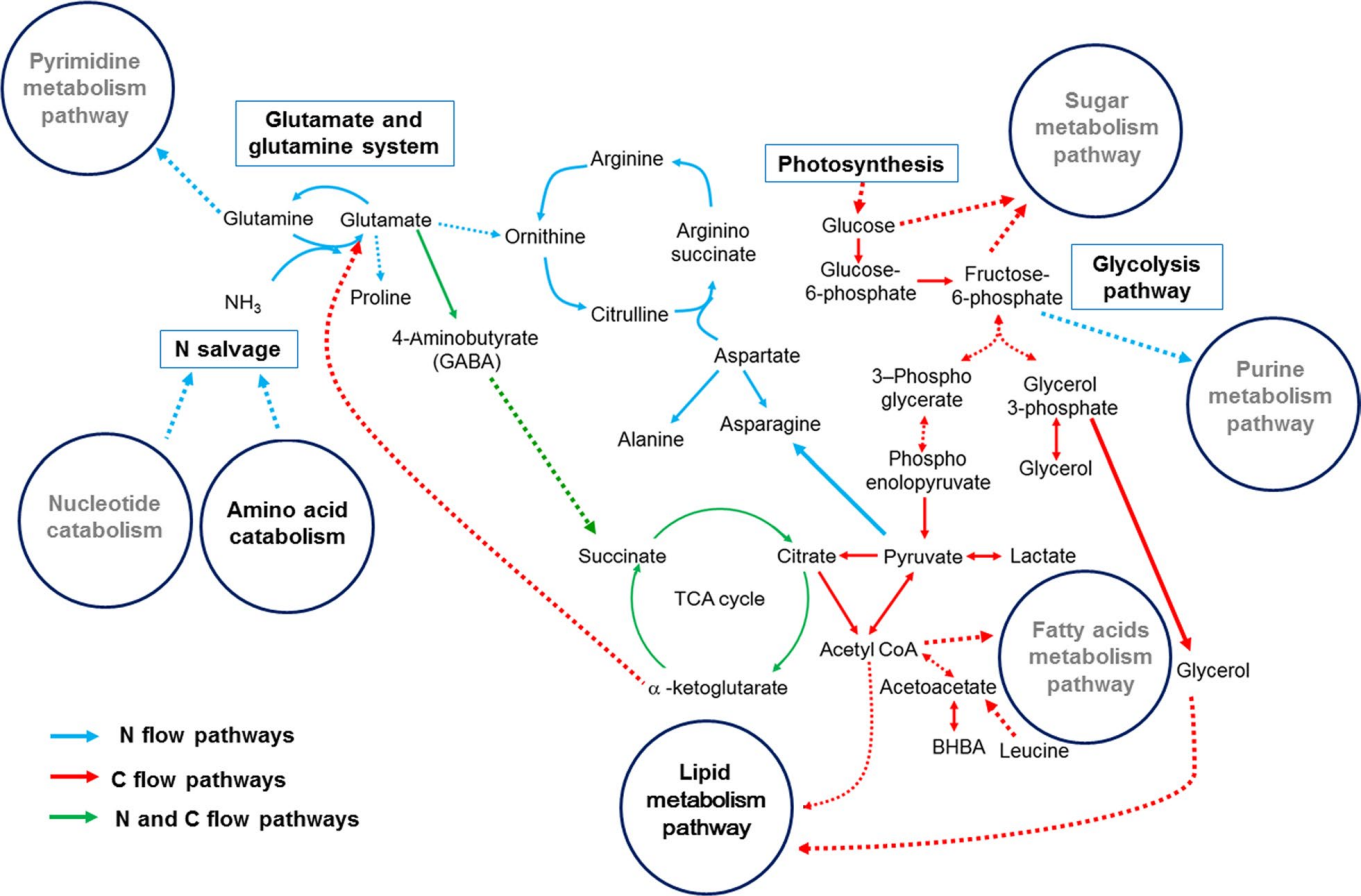
The distribution and contribution of nitrogen/carbon metabolism for lipid metabolism pathway. *Black words* the metabolic pathways which are related to lipid synthesis, *gray words* the metabolic pathways which are not related to lipid synthesis. *Blue arrow* N flow pathways, *red arrow* C flow pathways, *green arrow* N and C flow pathways. *Solid arrow* one step of metabolic flow, *dotted arrow* more than one step of metabolic flow

Thus, as shown in Fig. 10, we propose a scenario in which a series of metabolic pathways contribute to lipid metabolism. For high lipid-producing algae (e.g., C2), in N flow pathways, N salvaged from amino acid catabolism is assimilated and re-distributed via the glutamate–glutamine pathway and the corresponding transaminase pathways and stored as amino acids and intermediate molecules, including proline, GABA, alanine, arginine, and succinate, via the GABA pathway and the TCA cycle. In C flow pathways, C obtained either from photosynthesis or from glycolysis is re-distributed into C-containing compounds, such as G6P, F6P, PEP, lactate, citrate, BHBA, and leucine, via the GABA pathway and the TCA cycle. Further, as the re-distributed N is converted into N-containing molecules, leading to C/N disequilibrium, the excess C is re-distributed and diverted into storage lipid biosynthesis via the GABA pathway, glycolysis, and the TCA cycle. However, for medium or low lipid-producing algae (e.g., C1 or C3), in N flow pathways, N salvage is derived from nucleotide catabolism, assimilated and re-distributed via the glutamate–glutamine pathway and the corresponding transaminase pathways, and then stored as purine, pyrimidine, and intermediate molecules. In C flow pathways, the excess C obtained either from photosynthesis or from glycolysis is re-distributed and diverted into storage sugar molecules and fatty acids.

## Conclusions

In summary, the data obtained from the metabolic profile analysis and physiological analysis in this study indicate that the N/C assimilation and distribution pathways related to the glutamate–glutamine system, amino acid (GABA) catabolism and synthesis, and the TCA cycle and glycolysis contribute to the re-distribution of excess C for lipid biosynthesis. In addition, the current study provides useful information for enhancing lipid biosynthesis in microalgae via genetic manipulation of C/N metabolic pathways.

## Additional files

**Additional file 1: Table S1.** *Chlamydomonas* species used in this study. **Table S2.** Statistical summary. **Table S3.** Specific primers for gene quantitative real-time PCR analysis.

**Additional file 2: Appendix A.** Metabolon Platform. **Appendix B.** Statistical Terminology.

**Additional file 3.** Original Data Table.

**Additional file 4: Figure S1.** Lipid accumulation of 15 *Chlorella* strains grown in regular BG11 media at the stationary phase. 1. *Chlorella sorokiniana* C1; 2. *Chlorella* sp. C2; 3. *Chlorella sorokiniana* C3; 4. *Chlorella sorokiniana* C7; 5. *Chlorella* sp. A2; 6. FACHB1 (*Chlorella luteorividis*); 7, FACHB37 (*Chlorella vulgaris*); 8. FACHB960 (*Chlorella* sp.); 9. FACHB1068 (*Chlorella vulgaris*); 10. FACHB1216 (*Chlorella pyrenoidosa*); 11, FACHB1222 (*Chlorella pyrenoidosa*); 12. FACHB1227 (*Chlorella vulgaris*); 13. FACHB1552 (*Chlorella* sp.); 14. FACHB1568 (*Chlorella* sp.); 15. FACHB1580 (*Chlorella* sp.). FACHB1, FACHB37, FACHB960, FACHB1068, FACHB1216, FACHB1222, FACHB1227, FACHB1552, FACHB1568 and FACHB1580 were obtained from the Freshwater Algae Culture Collection of the Institute of Hydrobiology, Chinese Academy of Sciences. The expected lipid bands were marked using red box. The figure is representative of three replicated studies with similar findings. **Figure S2.** Physiological analysis of three *Chlorella* strains. A, growth curve of three *Chlorella* strains grown in regular BG11 medium; B, photos of three *Chlorella* strains grown in regular BG11 medium; C, total N content in culture medium during three *Chlorella* strains grown in regular BG11 medium; D, cell growth of three *Chlorella* strains following re-suspension in N- medium. All data points in the current and following figures represent the means and SD of three to five biological replicates (*t* test, *p* < 0.05). **Figure S3.** Overall trends analysis of lipid accumulation in three *Chlorella* strains. A, lipid accumulation analyzed by TLC during 0-9 d under N- treatment; asterisk symbol, glyceryl trioleate as loading standard; the expected lipid bands for further clarity were marked using red box; the figure is representative of three replicated studies with similar findings. B, lipid quantification during 2-9 d under N- treatment by using ImageJ (ver1.41, NIH), and the significance of the differences between the 2 d and other test values at 3-9 d in each panel was tested using a one-way ANOVA. *, *p* < 0.05. **Figure S4.** Lipid quantification of three *Chlorella* strains under N- treatment. Lipid content at 0 d, 1 d, 2 d and 6 d after N- treatment was determined according to Figure 1B by using ImageJ (ver1.41, NIH), and the significance of the differences between the C1 and other two strains was tested using a one-way ANOVA. *, *p* < 0.05. **Figure S5.** The global metabolic pathways of 220 detected compounds (black) and some undetected compounds (gray) of known identity in metabolome analysis in three *Chlorella* strains. Solid arrow, one step of metabolic flow; dotted arrow, more than one step of metabolic flow; blue dashed line box, the pathways related to nitrogen metabolism; red dashed line box, the pathways related to carbon metabolism. **Figure S6.** The relative enzyme activities verification of *Chlamydomonas reinhardtii* knock-out mutants deficient in glutamine synthetase (GS), glutamate synthase/NADH-dependent (NADH-GSN), glutamate synthase/Fd-dependent (Fd-GSN), aspartate aminotransferase (AST), alanine aminotransferase (ALT), pyruvate kinase (PK) and citrate synthase (CS). The control (background strain CC4533) value of each enzyme activity was set to 1 for easy comparison.

## Abbreviations

ALT: alanine aminotransferase
AST: aspartate aminotransferase
BHBA: 3-hydroxybutyrate
C: carbon
CLSM: confocal laser scanning microscopy
CS: citrate synthase
F6P: fructose-6-phosphate
FCM: flow cytometry
Fd-GSN: glutamate synthase/Fd-dependent
G6P: glucose-6-phosphate
GABA: gamma-aminobutyrate
GS: glutamine synthetase
N: nitrogen
N+: N-sufficient medium
N−: N-deficient medium
NADH-GSN: glutamate synthase/NADH-dependent
PEP: phosphoenolpyruvate
PK: pyruvate kinase
TCA: tricarboxylic acid
TLC: thin-layer chromatography
