# Supplementary material for: Comparative metabolic profiling of the lipid-producing green microalga Chlorella reveals that nitrogen and carbon metabolic pathways contribute to lipid metabolism
Source: Biotechnol Biofuels. 2017 Jun 15;10:153. doi: 10.1186/s13068-017-0839-4 (PMC5471736; doi:10.1186/s13068-017-0839-4)
Supplement: Supplementary file 1 — Additional file 1: Table S1. Chlamydomonas species used in this study. Table S2. Statistical summary. Table S3. Specific primers for gene quantitative real-time PCR analysis. [file 13068_2017_839_MOESM1_ESM.docx]

Table S1. *Chlamydomonas* species used in this study

| Strain Number | Common name of locus disrupted | Abbreviation |
| --- | --- | --- |
| LMJ.SG0182.007292 | Glutamate synthase / NADH-dependent | NADH-GSN |
| LMJ.SG0182.016146 | Aspartate aminotransferase | AST |
| LMJ.SG0182.017770 | Glutamine synthetase | GS |
| LMJ.SG0402.168249 | Citrate synthase | CS |
| LMJ.SG0402.72867 | Alanine aminotransferase | ALT |
| LMJ.SG0402.242303 | Pyruvate kinase | PK |
| LMJ.SG0402.185107 | Glutamate synthase / Fd-dependent | Fd-GSN |

Table S2. Statistical Summary

| **Statistical Comparisons** | | | | | | | | | | | | | | | | | | |
| --- | --- | --- | --- | --- | --- | --- | --- | --- | --- | --- | --- | --- | --- | --- | --- | --- | --- | --- |
| **Two-Way ANOVA Contrasts** | **Medium 1 Medium 0** | **Medium 2 Medium 0** | | **Medium 6 Medium 0** | | | **High 1 High 0** | | **High 2 High 0** | | **High 6 High 0** | | | **Low 1 Low 0** | | **Low 2 Low 0** | | **Low 6 Low 0** |
| **Total biochemicals *p*≤0.05** | 109 | 143 | | 148 | | | 124 | | 126 | | 162 | | | 110 | | 120 | | 154 |
| **Biochemicals  (↑↓)** | **73 \| 36** | **92 \| 51** | | **80 \| 68** | | | **72 \| 52** | | **76 \| 50** | | **67 \| 95** | | | **40 \| 70** | | **55 \| 65** | | **77 \| 77** |
| **Total biochemicals 0.05<*p*<0.10** | 14 | 9 | | 11 | | | 11 | | 10 | | 7 | | | 5 | | 10 | | 16 |
| **Biochemicals  (↑↓)** | **7 \| 7** | **5 \| 4** | | **6 \| 5** | | | **5 \| 6** | | **2 \| 8** | | **5 \| 2** | | | **3 \| 2** | | **6 \| 4** | | **6 \| 10** |
| **ANOVA Contrasts cont.** | **Medium 0 Low 0** | | **High 0 Low 0** | | **Medium 1 Low 1** | | | **High 1 Low 1** | | **Medium 2 Low 2** | | **High 2 Low 2** | | | **Medium 6 Low 6** | | **High 6 Low 6** | |
| **Total biochemicals *p*≤0.05** | 100 | | 171 | | 105 | | | 153 | | 91 | | 160 | | | 77 | | 154 | |
| **Biochemicals  (↑↓)** | **40 \| 60** | | **93 \| 78** | | **75 \| 30** | | | **96 \| 57** | | **70 \| 21** | | **94 \| 66** | | | **44 \| 33** | | **60 \| 94** | |
| **Total biochemicals 0.05<*p*<0.10** | 9 | | 6 | | 12 | | | 6 | | 12 | | 7 | | | 17 | | 4 | |
| **Biochemicals  (↑↓)** | **5 \| 4** | | **2 \| 4** | | **7 \| 5** | | | **3 \| 3** | | **6 \| 6** | | **5 \| 2** | | | **8 \| 9** | | **1 \| 3** | |
| **ANOVA Main Effects** | **Lipid Main Effect** | | | | | **Time Main Effect** | | | | | | | **Lipid : Time Interaction** | | | | | |
| **Total biochemicals *p*≤0.05** | 202 | | | | | 194 | | | | | | | 178 | | | | | |
| **Total biochemicals 0.05<*p*<0.10** | 5 | | | | | 10 | | | | | | | 12 | | | | | |

Table S3. Specific primers for gene quantitative real-time PCR analysis

| Gene | Primer sequence (5`-3`) | *C. reinhardtii* database matches [Pytozome locus name or GenBank Accession Number*] |
| --- | --- | --- |
| ALT | \| GGGCAAGGAGATCATTTTCA \| \| --- \| \| CTAGGATCTTCTTGGCACGC \| | Cre10.g451950 |
| NADH-GSN | \| AGATTCTGTAGATTACGGTTTCGC \| \| --- \| \| CACAAACTACTCAACACAGCACAG \| | Cre13.g592200 |
| Fd-GSN | \| GACCAAGCTGGAGGACTACG \| \| --- \| \| CTGCTGGTAGGCCTTGTAGG \| | Cre12.g514050 |
| AST1 | CTTGCCTGGTGGTGGTATG  GCCATACTCGGAAGCCAAT | Cre09.g387726 |
| AST2 | TTGACTGGCCTGGTCTGAG  CCATGCAGTCCAGCACTCT | Cre01.g051800 |
| AST3 | AAGGAGCTGTTCGAGGAGTG  GAAGCTGAACATGCCGATCT | Cre02.g097900 |
| AST4 | CGATATCCCCAGGATTAGGAA  TACGCATGCAACACATTCATT | Cre06.g257950 |
| GS1 | ACCAGTCGGGGATTTCTTCT  ATCCACACATACTCGGCACA | Cre02.g113200 |
| GS2 | AGGGCATGATCTTCAACGAG  AGGATGCAGTCCGAGTTGTT | Cre12.g530650 |
| GS3 | TTCTGGTCCTCTGCGACAC  TGATGGCGTACTCTGCTC | Cre03.g207250 |
| CS | GGTGAAAATGGAGTGCATGA  AGTGCAACGTCTGCGACATA | Cre12.g514750 |
| PK1 | AAGACCAAGGTGGTTTGCAC  CGAAGGTTGTCAAGGGTCTC | Cre.2797 |
| PK2 | GACAGCAAAGGAACATGCAA  TTTGACCGGGAAACTAATGC | Cre06.g280950 |
| PK3 | CACGAACGTGACGCTCTCTA  AAGTTGAAGCGGAGCACATT | Cre05.g234700 |
| PK4 | CAGTCCCGTACTGTGGAGGT  ATAGCGATGCGCAGGTTATC | Cre02.g147900 |
| PK5 | GGACACCGGATTTGAGATTG  GAATCCGGAAGTTGAACAGC | cre:CHLREDRAFT_196892 |
| CBLP | GCTGTGGGACCTGGCTGA  CCTTCTTGCTGGTGATGTTG | g6364 |

*Pytozome locus name database is available under <http://www.phytozome.net/search.php?show=text&method=Org_Creinhardtii>.
