# Supplementary material for: Comparative metabolic profiling of the lipid-producing green microalga Chlorella reveals that nitrogen and carbon metabolic pathways contribute to lipid metabolism
Source: Biotechnol Biofuels. 2017 Jun 15;10:153. doi: 10.1186/s13068-017-0839-4 (PMC5471736; doi:10.1186/s13068-017-0839-4)
Supplement: Supplementary file 2 — Additional file 2: Appendix A. Metabolon Platform. Appendix B. Statistical Terminology. [file 13068_2017_839_MOESM2_ESM.docx]

**Appendix A: Metabolon Platform**

**Sample Accessioning:** Each sample received was accessioned into the Metabolon LIMS system and was assigned by the LIMS a unique identifier, which was associated with the original source identifier only. This identifier was used to track all sample handling, tasks, results *etc.* The samples (and all derived aliquots) were bar-coded and tracked by the LIMS system. All portions of any sample were automatically assigned their own unique identifiers by the LIMS when a new task was created; the relationship of these samples was also tracked. All samples were maintained at -80 ºC until processed.

**Sample Preparation:** The sample preparation process was carried out using the automated MicroLab STAR® system from Hamilton Company. Recovery standards were added prior to the first step in the extraction process for QC purposes. Sample preparation was conducted using a proprietary series of organic and aqueous extractions to remove the protein fraction while allowing maximum recovery of small molecules. The resulting extract was divided into two fractions; one for analysis by UPLC-MS/MS and one for analysis by GC/MS. Samples were placed briefly on a TurboVap® (Zymark) to remove the organic solvent. Each sample was then frozen and dried under vacuum. Samples were then prepared for the appropriate instrument, either LC/MS or GC/MS.

**QA/QC:** For QA/QC purposes, a number of additional samples are included with each day’s analysis. Furthermore, a selection of QC compounds is added to every sample, including those under test. These compounds are carefully chosen so as not to interfere with the measurement of the endogenous compounds. Tables 1 and 2 describe the QC samples and compounds. These QC samples are primarily used to evaluate the process control for each study as well as aiding in the data curation.

**Table A1:** Description of Metabolon QC Samples

| **Type** | **Description** | **Purpose** |
| --- | --- | --- |
| MTRX | Large pool of human plasma maintained by Metabolon that has been characterized extensively. | Assure that all aspects of Metabolon process are operating within specifications. |
| CMTRX | Pool created by taking a small aliquot from every customer sample. | Assess the effect of a non-plasma matrix on the Metabolon process and distinguish biological variability from process variability. |
| PRCS | Aliquot of ultra-pure water | Process Blank used to assess the contribution to compound signals from the process. |
| SOLV | Aliquot of solvents used in extraction. | Solvent blank used to segregate contamination sources in the extraction. |

**Table A2:** Metabolon QC Standards

| **Type** | **Description** | **Purpose** |
| --- | --- | --- |
| DS | Derivatization Standard | Assess variability of derivatization for GC/MS samples. |
| IS | Internal Standard | Assess variability and performance of instrument. |
| RS | Recovery Standard | Assess variability and verify performance of extraction and instrumentation. |

**Liquid chromatography/Mass Spectrometry (LC/MS, LC/MS^2^**): The LC/MS portion of the platform was based on a Waters ACQUITY UPLC and a Thermo-Finnigan LTQ mass spectrometer, which consisted of an electrospray ionization (ESI) source and linear ion-trap (LIT) mass analyzer. The sample extract was split into two aliquots, dried, then reconstituted in acidic or basic LC-compatible solvents, each of which contained 11 or more injection standards at fixed concentrations. One aliquot was analyzed using acidic positive ion optimized conditions and the other using basic negative ion optimized conditions in two independent injections using separate dedicated columns. Extracts reconstituted in acidic conditions were gradient eluted using water and methanol both containing 0.1% Formic acid, while the basic extracts, which also used water/methanol, contained 6.5mM Ammonium Bicarbonate. The MS analysis alternated between MS and data-dependent MS^2^ scans using dynamic exclusion.

**Ultrahigh Performance Liquid Chromatography/Mass Spectroscopy (UPLC/MS/MS):** The LC/MS portion of the platform was based on a Waters ACQUITY ultra-performance liquid chromatography (UPLC) and a Thermo Scientific Q-Exactive high resolution/accurate mass spectrometer interfaced with a heated electrospray ionization (HESI-II) source and Orbitrap mass analyzer operated at 35,000 mass resolution. The sample extract was dried then reconstituted in acidic or basic LC-compatible solvents, each of which contained 8 or more injection standards at fixed concentrations to ensure injection and chromatographic consistency. One aliquot was analyzed using acidic positive ion optimized conditions and the other using basic negative ion optimized conditions in two independent injections using separate dedicated columns. Extracts reconstituted in acidic conditions were gradient eluted using water and methanol containing 0.1% formic acid, while the basic extracts, which also used water/methanol, contained 6.5mM Ammonium Bicarbonate. The MS analysis alternated between MS and data-dependent MS^2^ scans using dynamic exclusion, and the scan range was from 80-1000 *m/z*. Raw data files are archived and extracted as described below.

**Gas chromatography/Mass Spectrometry (GC/MS**): The samples destined for GC/MS analysis were re-dried under vacuum desiccation for a minimum of 24 hours prior to being derivatized under dried nitrogen using bistrimethyl-silyl-triflouroacetamide (BSTFA). The GC column was 5% phenyl and the temperature ramp is from 40° to 300° C in a 16 minute period. Samples were analyzed on a Thermo-Finnigan Trace DSQ fast-scanning single-quadrupole mass spectrometer using electron impact ionization. The instrument was tuned and calibrated for mass resolution and mass accuracy on a daily basis. The information output from the raw data files was automatically extracted as discussed below.

**Bioinformatics:** The informatics system consisted of four major components, the Laboratory Information Management System (LIMS), the data extraction and peak-identification software, data processing tools for QC and compound identification, and a collection of information interpretation and visualization tools for use by data analysts. The hardware and software foundations for these informatics components were the LAN backbone, and a database server running Oracle 10.2.0.1 Enterprise Edition.

**LIMS:** The purpose of the Metabolon LIMS system was to enable fully auditable laboratory automation through a secure, easy to use, and highly specialized system. The scope of the Metabolon LIMS system encompasses sample accessioning, sample preparation and instrumental analysis and reporting and advanced data analysis. All of the subsequent software systems are grounded in the LIMS data structures. It has been modified to leverage and interface with the in-house information extraction and data visualization systems, as well as third party instrumentation and data analysis software.

**Data Extraction and Compound Identification:** Raw data was extracted, peak-identified and QC processed using Metabolon’s hardware and software. These systems are built on a web-service platform utilizing Microsoft’s .NET technologies, which run on high-performance application servers and fiber-channel storage arrays in clusters to provide active failover and load-balancing (2). Compounds were identified by comparison to library entries of purified standards or recurrent unknown entities. Metabolon maintains a library based on authenticated standards that contains the retention time/index (RI), mass to charge ratio (*m/z)*, and chromatographic data (including MS/MS spectral data) on all molecules present in the library. Furthermore, biochemical identifications are based on three criteria: retention index within a narrow RI window of the proposed identification, nominal mass match to the library +/- 0.4 amu, and the MS/MS forward and reverse scores between the experimental data and authentic standards. The MS/MS scores are based on a comparison of the ions present in the experimental spectrum to the ions present in the library spectrum. While there may be similarities between these molecules based on one of these factors, the use of all three data points can be utilized to distinguish and differentiate biochemicals. More than 3500 commercially available purified standard compounds have been acquired and registered into LIMS for distribution to both the LC-MS and GC-MS platforms for determination of their analytical characteristics.

**Curation:** A variety of curation procedures were carried out to ensure that a high quality data set was made available for statistical analysis and data interpretation. The QC and curation processes were designed to ensure accurate and consistent identification of true chemical entities, and to remove those representing system artifacts, mis-assignments, and background noise. Metabolon data analysts use proprietary visualization and interpretation software to confirm the consistency of peak identification among the various samples. Library matches for each compound were checked for each sample and corrected if necessary.

**Normalization:** For studies spanning multiple days, a data normalization step was performed to correct variation resulting from instrument inter-day tuning differences. Essentially, each compound was corrected in run-day blocks by registering the medians to equal one (1.00) and normalizing each data point proportionately (termed the “block correction”; Figure 1). For studies that did not require more than one day of analysis, no normalization is necessary, other than for purposes of data visualization.

**Figure A1:** Visualization of Data Normalization

**Statistical Calculation:** For many studies, two types of statistical analysis are usually performed: (1) significance tests and (2) classification analysis. (1) For pair-wise comparisons we typically perform Welch’s t-tests and/or Wilcoxon’s rank sum tests. For other statistical designs we may perform various ANOVA procedures (e.g., repeated measures ANOVA). (2) For classification we mainly use random forest analyses. Random forests give an estimate of how well we can classify *individuals* in a *new* data set into each group, in contrast to a t-test, which tests whether the unknown means for two populations are different or not. Random forests create a set of classification trees based on continual sampling of the experimental units and compounds. Then each observation is classified based on the majority votes from all the classification trees. Statistical analyses are performed with the program “R” <http://cran.r-project.org/>.

**Appendix B: Statistical Terminology**

***t*-tests:** *t*-tests test whether the unknown means for two populations are different or not. The *p*-value gives the amount evidence that the population means are different based on the data (through the *t*-statistic). The smaller the *p*-value, the more evidence we have that the population means are different. Often, a significance level of 0.05 is used. When the *p*-value is less than 0.05, we have enough evidence to conclude that the population means are different (“statistical significance”). The level of 0.05 is the false positive rate. This means that 5% of the time, the *t*-test would incorrectly conclude the population means are different when they are the actually the same.

***q*-values**: The level of 0.05 is the false positive rate when there is one test. However, for a large number of tests we need to account for false positives. If the data were simply random noise, approximately 5% of the *p*-values would be less than 0.05, 10% of the *p*-values would be less than 0.10, etc. Thus, even if the data were only random noise, we would get approximately 10 “significant” results out of 200 compounds when the false positive rate is 0.05.

There are different methods to correct for multiple testing. The oldest methods are family-wise error rate adjustments (Bonferroni, Tukey, etc.), but these tend to be extremely conservative for a very large number of tests. With gene arrays, using the False Discovery Rate (FDR) is more common. The family-wise error rate adjustments give one a high degree of confidence that there are *zero* false discoveries. However, with FDR methods, one can allow for a small number of false discoveries. The FDR for a given set of compounds can be estimated using the *q*-value (see Storey J and Tibshirani R. (2003) Statistical significance for genomewide studies. *Proc. Natl. Acad. Sci. USA* **100**: 9440-9445).

In order to interpret the *q*-value, first sort the data by the *p*-value then choose the cutoff for significance (typically *p*<0.05). The *q*-value gives the false discovery rate for the selected list (i.e., an estimate of the proportion of false discoveries for the list of compounds whose *p*-value is below the cutoff for significance). For Table 1 below, if the whole list is declared significant, then the false discovery rate is approximately 10%. If everything from Compound 079 and above is declared significant, then the false discovery rate is approximately 2.5%.

**Table B1:** Example of *q*-value interpretation

***Random Forest:*** Random forest is a supervised classification technique based on an ensemble of decision trees (see Breiman. 2001. *Machine Learning*. 45:5, for the original description; Goldstein et al. 2010. *BMC Genetics*. 11:49, for additional information). For a given decision tree, a random subset of the data with identifying true class information is selected to build the tree (“bootstrap sample” or “training set”), and then the remaining data, the “out-of-bag” (OOB) variables, are passed down the tree to obtain a class prediction for each sample. This process is repeated thousands of times to produce the forest. The final classification of each sample is determined by computing the class prediction frequency (“votes”) for the OOB variables over the whole forest. For example, suppose the random forest consists of 50,000 trees and that 25,000 trees had a prediction for sample 1. Of these 25,000, suppose 15,000 trees classified the sample as belonging to Group A and the remaining 10,000 classified it as belonging to Group B. Then the votes are 0.6 for Group A and 0.4 for Group B, and hence the final classification is Group A. This method is unbiased since the prediction for each sample is based on trees built from a subset of samples that do not include that sample. When the full forest is grown, the class predictions are compared to the true classes, generating the “OOB error rate” as a measure of prediction accuracy. Thus, the prediction accuracy is an unbiased estimate of how well one can predict sample class in a new data set.

To determine which variables (biochemicals) make the largest contribution to the classification, a “variable importance” measure is computed. We use the “Mean Decrease Accuracy” (MDA) as this metric. The MDA is determined by randomly permuting a variable, running the observed values through the trees, and then reassessing the prediction accuracy. If a variable is not important, then this procedure will have little change in the accuracy of the class prediction (permuting random noise will give random noise). By contrast, if a variable is important to the classification, the prediction accuracy will drop after such a permutation, which we record as the MDA. Thus, the random forest analysis provides an “importance” rank ordering of biochemicals; we typically output the top 30 biochemicals in the list as potentially worthy of further investigation.
