# Supplementary figures and images for: Comparative metabolic profiling of the lipid-producing green microalga Chlorella reveals that nitrogen and carbon metabolic pathways contribute to lipid metabolism
Source: Biotechnol Biofuels. 2017 Jun 15;10:153. doi: 10.1186/s13068-017-0839-4 (PMC5471736; doi:10.1186/s13068-017-0839-4)

**Fig. S1**

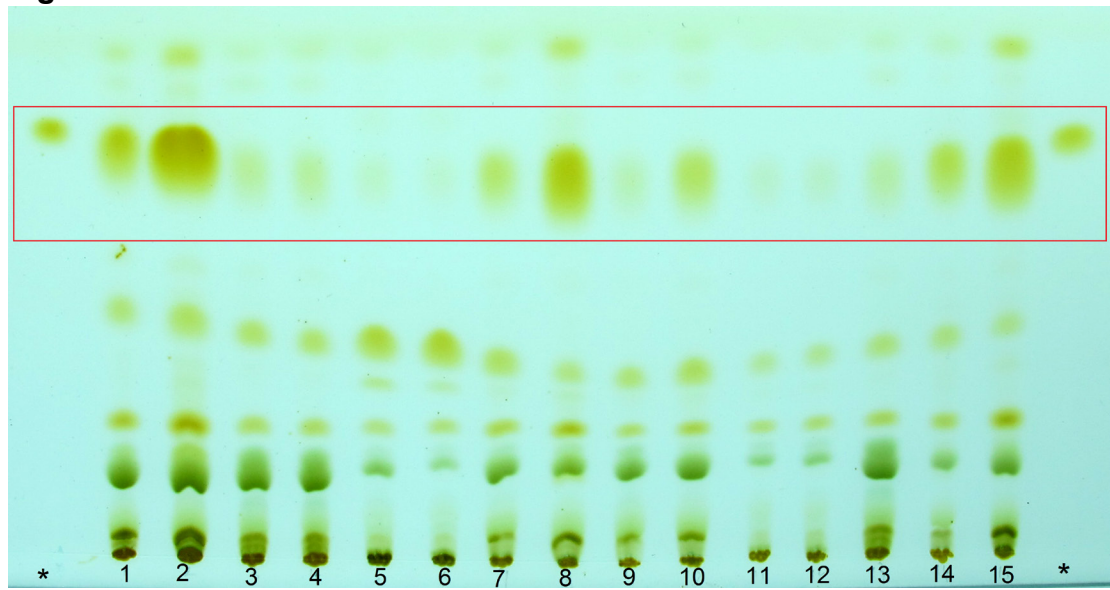

**Fig. S2**

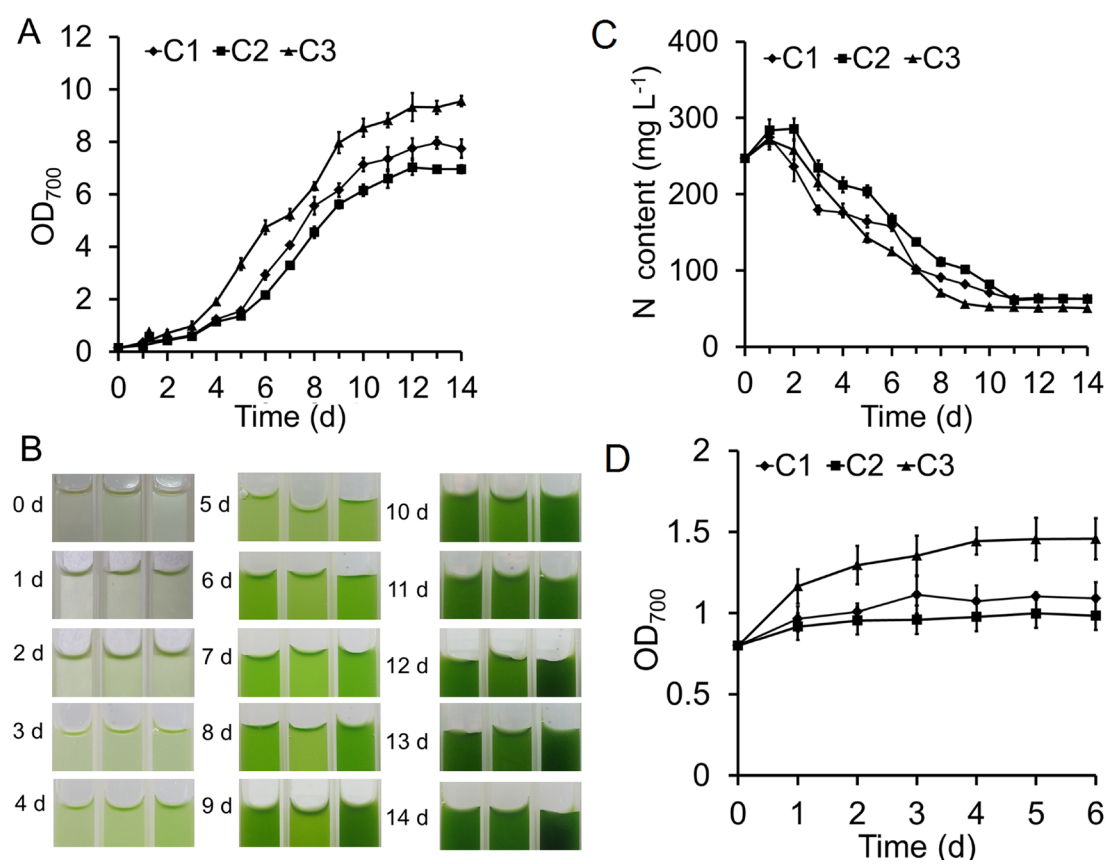

**Fig. S3**

**A**

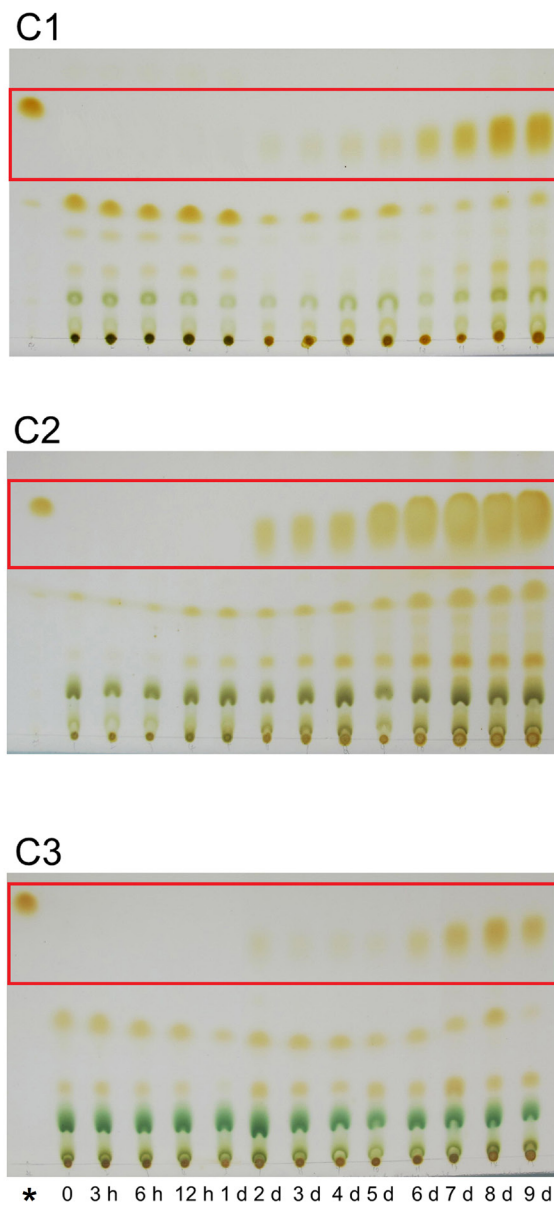

**B**

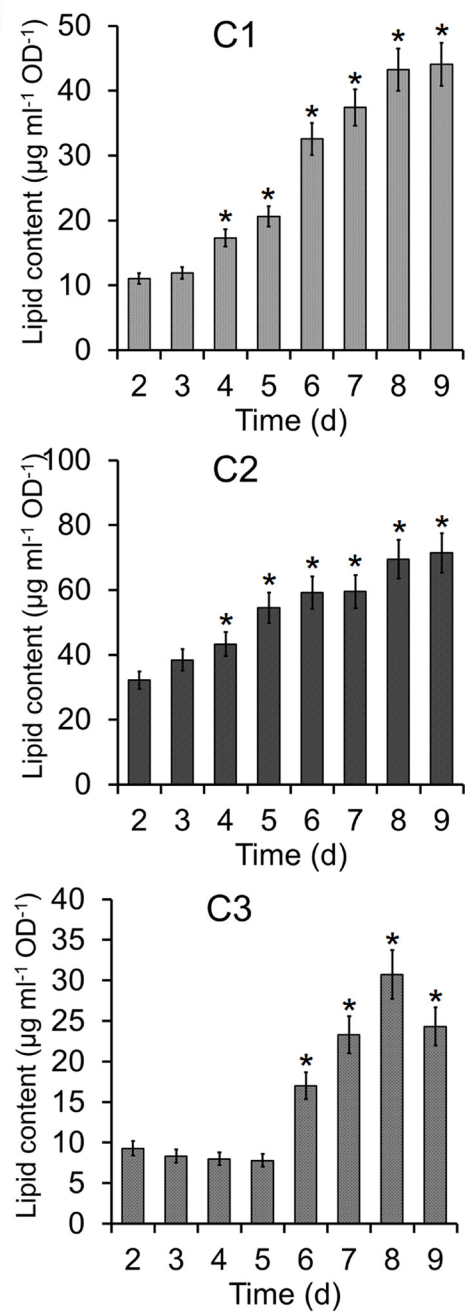

**Fig. S4**

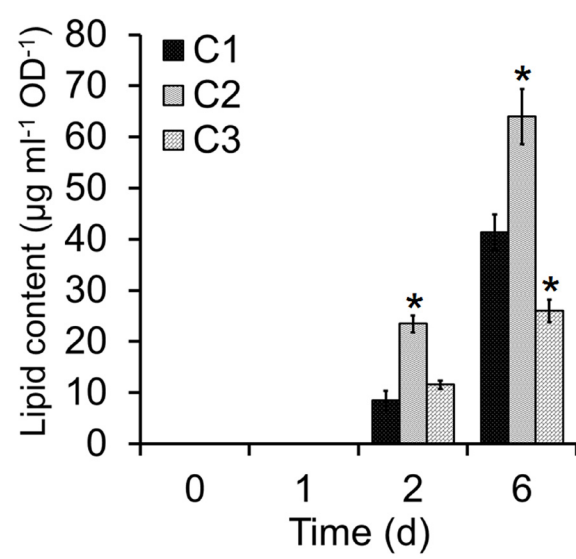

[illegible]

Fig. S6

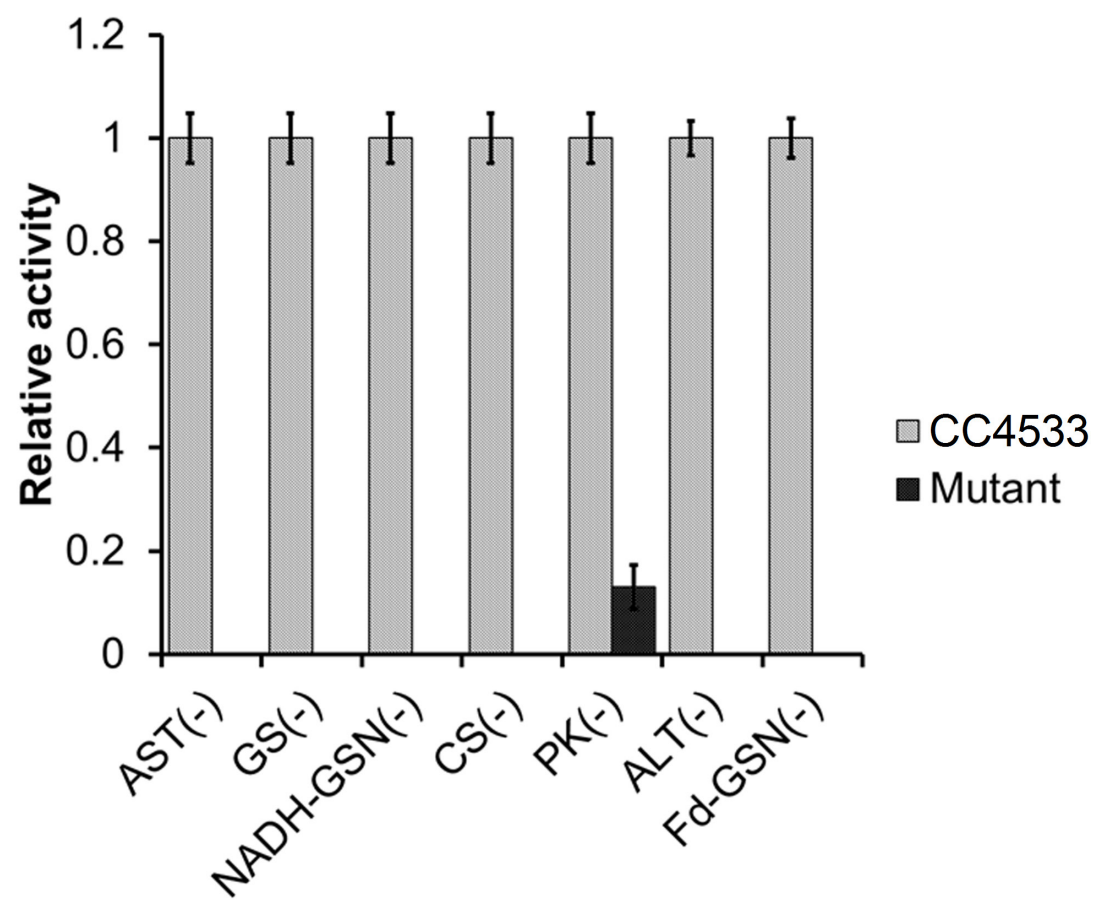

Supplement: Supplementary file 4 — Additional file 4: Figure S1. Lipid accumulation of 15 Chlorella strains grown in regular BG11 media at the stationary phase. 1. Chlorella sorokiniana C1; 2. Chlorella sp. C2; 3. Chlorella sorokiniana C3; 4. Chlorella sorokiniana C7; 5. Chlorella sp. A2; 6. FACHB1 (Chlorella luteorividis); 7, FACHB37 (Chlorella vulgaris); 8. FACHB960 (Chlorella sp.); 9. FACHB1068 (Chlorella vulgaris); 10. FACHB1216 (Chlorella pyrenoidosa); 11, FACHB1222 (Chlorella pyrenoidosa); 12. FACHB1227 (Chlorella vulgaris); 13. FACHB1552 (Chlorella sp.); 14. FACHB1568 (Chlorella sp.); 15. FACHB1580 (Chlorella sp.). FACHB1, FACHB37, FACHB960, FACHB1068, FACHB1216, FACHB1222, FACHB1227, FACHB1552, FACHB1568 and FACHB1580 were obtained from the Freshwater Algae Culture Collection of the Institute of Hydrobiology, Chinese Academy of Sciences. The expected lipid bands were marked using red box. The figure is representative of three replicated studies with similar findings. Figure S2. Physiological analysis of three Chlorella strains. A, growth curve of three Chlorella strains grown in regular BG11 medium; B, photos of three Chlorella strains grown in regular BG11 medium; C, total N content in culture medium during three Chlorella strains grown in regular BG11 medium; D, cell growth of three Chlorella strains following re-suspension in N- medium. All data points in the current and following figures represent the means and SD of three to five biological replicates (t test, p < 0.05). Figure S3. Overall trends analysis of lipid accumulation in three Chlorella strains. A, lipid accumulation analyzed by TLC during 0-9 d under N- treatment; asterisk symbol, glyceryl trioleate as loading standard; the expected lipid bands for further clarity were marked using red box; the figure is representative of three replicated studies with similar findings. B, lipid quantification during 2-9 d under N- treatment by using ImageJ (ver1.41, NIH), and the significance of the differences between the 2 d and other test va [file 13068_2017_839_MOESM4_ESM.pdf]
